# In vitro evolution and whole genome analysis to study chemotherapy drug resistance in haploid human cells

**DOI:** 10.1038/s41598-024-63943-7

**Published:** 2024-06-18

**Authors:** Juan Carlos Jado, Michelle Dow, Krypton Carolino, Adam Klie, Gregory J. Fonseca, Trey Ideker, Hannah Carter, Elizabeth A. Winzeler

**Affiliations:** 1 Division of Host-Microbe Systems & Therapeutics, Department of Pediatrics, University of California, San Diego, Gilman Dr., La Jolla, CA 92093 USA; 2 Department of Medicine, Division of Medical Genetics, University of California San Diego, La Jolla, CA 92093 USA; 3 Bioinformatics and Systems Biology Graduate Program, University of California San Diego, La Jolla, CA 92093 USA; 4 Health Science, Department of Biomedical Informatics, School of Medicine, University of California San Diego, La Jolla, CA 92093 USA; 5 Division of Biological Sciences, University of California San Diego, La Jolla, CA 92093 USA; 6 Department of Medicine, Meakins-Christie Laboratories, McGill University Health Centre, 1001 Decaire Blvd, Montreal, QC H4A 3J1 Canada; 7 Moores Cancer Center, University of California San Diego, La Jolla, CA 92093 USA

**Keywords:** Chemical genetics, Drug development

## Abstract

In vitro evolution and whole genome analysis has proven to be a powerful method for studying the mechanism of action of small molecules in many haploid microbes but has generally not been applied to human cell lines in part because their diploid state complicates the identification of variants that confer drug resistance. To determine if haploid human cells could be used in MOA studies, we evolved resistance to five different anticancer drugs (doxorubicin, gemcitabine, etoposide, topotecan, and paclitaxel) using a near-haploid cell line (HAP1) and then analyzed the genomes of the drug resistant clones, developing a bioinformatic pipeline that involved filtering for high frequency alleles predicted to change protein sequence, or alleles which appeared in the same gene for multiple independent selections with the same compound. Applying the filter to sequences from 28 drug resistant clones identified a set of 21 genes which was strongly enriched for known resistance genes or known drug targets (*TOP1, TOP2A*, *DCK*, *WDR33, SLCO3A1*). In addition, some lines carried structural variants that encompassed additional known resistance genes (*ABCB1, WWOX and RRM1*). Gene expression knockdown and knockout experiments of 10 validation targets showed a high degree of specificity and accuracy in our calls and demonstrates that the same drug resistance mechanisms found in diverse clinical samples can be evolved, discovered and studied in an isogenic background.

## Introduction

In human cells, methods for discovering genes that play a role in drug resistance or which encode drug targets, especially for poorly characterized compounds, such as natural products, are limited. Genome-wide CRISPR-*Cas9* knockdown experiments^1–3^ in the presence of a drug are useful to broadly implicate relevant genes, but cannot readily reveal critical gain-of-function, single nucleotide alleles, such as imatinib-resistance conferring mutations in BCR-Abl. Discovering common alleles in whole genome sequences of tumors from cohorts of patients that have relapsed after drug treatment requires very large datasets and is complicated by patient heterogeneity. Furthermore, such studies also cannot be used on experimental therapies.

Work in other organisms has shown that in vitro evolution and whole genome analysis (IVIEWGA) is a powerful method to discover both a comprehensive set of drug resistance alleles, as well as the targets of compounds with unknown mechanisms of action^4,5^. In this method, clonal or near clonal organisms are isolated and then clones are subjected to increasing levels of a drug that inhibits growth. After selection, the organism is cloned again. The genomes of resistant clones are then compared to the sensitive parent clone using next generation sequencing (NGS) methods. In organisms such as *Saccharomyces cerevisiae*^6^, *Plasmodium falciparum*^4,5^, Mycobacteria^7^, Trypanosomes^6^, and *Acinetobacter baumannii*^8^ this method has been used to comprehensively discover resistance conferring variants. Surprisingly, the data shows that typically only a small number of de novo variants are detected after evolution. If multiple selections are performed on independent clones, the same resistance gene will appear repeatedly, although often appearing with different alleles, providing a high level of statistical confidence that the allele has not arisen by chance.

Many of the organisms on which IVIEWGA has been used with success have both haploid and diploid phases of their lifecycle, which means that selections can be performed in a haploid stage. Selecting for resistant clones in a haploid organism greatly simplifies analysis as a driver resistance allele will approach 100% frequency. In addition, for loss of function alleles, only one mutation is needed (versus both copies). Although metazoans are all diploid, haploid human cell lines are nevertheless available: HAP1, is a human chronic myelogenous leukemia (CML)-derived cell line that is completely haploid except for a 30 megabase fragment of chromosome 15 ^9^. HAP1 has been used for genetic studies because mutated phenotypes are immediately exposed^10–15^.

Using five different anticancer drugs (Doxorubicin, Gemcitabine, Etoposide, Topotecan, and Paclitaxel) as examples, we show that in vitro evolution in HAP1 cells can be used to identify the molecular basis of drug resistance in human-derived cells. Through our unbiased analysis of evolved clones, we detect a limited number of genes that acquire SNVs or CNVs after prolonged, sublethal exposure to our selected xenobiotics. We further demonstrate the power of the approach by using shRNAs and CRISPR-*Cas9* to downregulate or reintroduce selected alleles and demonstrate that this confers resistance or sensitivity to the drug which elicited the evolved genomic change. Our work has implications for clinical intervention strategies to prevent the emergence of drug resistance and tumor recurrence through gene mutations acquired through DNA damage from chemotherapeutics or natural variants which exist and persist from the heterogenous tumor cell environment.

## Results

### Selection of compounds for resistance studies

**Figure 1:**
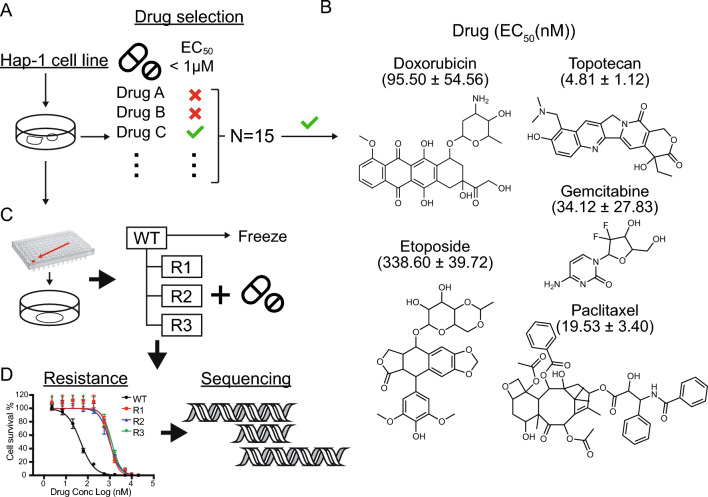
Experimental workflow. (**A**) Chemotherapy drug evaluation. EC_50_ dose response assays were performed on 16 different chemotherapeutic agents (Table S1). Only drugs to which HAP1 cells were sensitive (EC_50_ value below 1 µM) were considered for IVIEWGA. (**B**) Chemical structures of the chemotherapy agents ultimately used for IVIEWGA. EC_50_ values are presented as the mean ± s.e.m., for n = 3 biological replicates and n = 8 with technical replicates per concentration point. (**C**) Clone selection. To ensure a homogenous genetic background limiting dilution cloning was used to isolate individual cells prior to drug selection. For each drug three independent selections were performed. Resistance was confirmed using dose–response assays (**D**) Drug resistance was achieved in 7–30 weeks approximately (49 and 210 generations). The parental cell line and the drug resistant lines were then sequenced.

Our previous studies have shown that evolution works best with potent inhibitors of cell growth. We therefore sought to identify proof-of-concept compounds which would show 50% growth inhibition at concentrations of less than 10 μM and ideally less than 1 μM. To do this, 16 readily-available compounds were tested for the ability to reduce ATP levels (measured using CellTiterGlo) in HAP1 cells using a 48-h dose response assay. Although sometimes longer incubation times can be used in dose response assays, especially for slow growing cell lines, 48 h provided a good signal to noise ratio for our purposes and longer incubation times were not considered. The 16 compounds we tested had previously been tested against *S. cerevisiae*. Some were older drugs derived from natural products (e.g. rapamycin, topotecan) known to have activity against many different eukaryotic cell lines. Others were modern cancer drugs, including the kinase inhibitors, lisitinib, imatinib and sorafenib, which are predicted to act only against specific targets in specific cell lines. We include a separate section on the types of drugs applicable to this method in the discussion. Only five drugs showed EC_50_ values between 5 and 340 nM (Fig. 1A,B, Table S1). These included doxorubicin (DOX, EC_50_ = 95.50 ± 54.56 nM), also known as adriamycin, an anthracycline antibiotic that works by inhibiting topoisomerase II alpha (*TOP2A*)^16,17^; gemcitabine (GEM, EC_50_ = 34.12 ± 27.83 nM), a synthetic pyrimidine nucleoside prodrug that is used against a variety of hematopoietic malignancies^18–20^; etoposide (ETP, EC_50_ = 338.60 ± 39.72 nM), a semisynthetic derivative of podophyllotoxin that also targets *TOP2A* and prevents re-ligation of the double-stranded DNA^21^; paclitaxel (PTX, EC_50_ = 19.43 ± 3.40 nM) also known as taxol, an effective anticancer agent that targets tubulin, perturbing the cytoskeleton and causing M phase cell-cycle arrest^22^, and topotecan (TPT, EC_50_ = 4.81 ± 1.12 nM), a semisynthetic water-soluble derivative of camptothecin that inhibits topoisomerase I (*TOP1*)^23^. Our HAP1 EC_50_ values were similar to those previously reported for other CML cell lines (www.cancerrxgene.org^24,25^) with the exception of etoposide, which appeared more effective in HAP1 cells (EC_50_ = 338.6 ± 39.72 nM) relative to other CML cell lines (> 1 µM in BV-173, KU812, EM-2, MEG-01, JURL-MK1, KCL-22, RPMI-8866, LAMA-84, K-562). As described below, ETP resistance seemed to be associated with CNVs which could potentially explain some of the difference between published values for diploid lines and these haploid cells.

### Evolution of resistance is readily achieved for all compounds

Our next objective was to create drug resistant lines. Although we have had difficulty creating resistant lines for some drugs in some species (“irresistibles”^26^), there is precedent for resistance to the drugs included here^27–29^. To reduce the possibility of selecting mutations that were present in a non-homogenous population of HAP1 cells and to facilitate later genomic analysis, we first cloned the cells. This was accomplished by diluting cells to an average density of ~ 0.5 cells per well in a poly-l-lysine treated 96-well plate (Fig. 1C) and then picking clones from wells that contained single colonies. Selections were initiated with different parent clones for the different drug-specific replicates (Fig. 1C, Fig. S1).

To create drug resistant clones, cells were first grown in tissue culture dishes (reaching 60–80% semi-confluence) in the presence of sublethal concentrations of each drug. Most cell lines (DOX, GEM, TPT and PTX resistant clones) were then subjected to a lethal concentration (~ 3–5 × EC_50_ value), killing more than 95% of the cells. Treatment was removed until cells reached semi-confluence again (doubling every 22 h^30^) whereupon drug at ~ the EC_95_ value was reapplied. Alternatively, for ETP-resistant clones, a stepwise selection method was used whereby cells were repeatedly exposed to a concentration that killed around 50% of the cell population. Drug concentration was increased by 5–10% every 5 days while keeping the growth rate at 50% of untreated culture. Although others have used mutagenesis^31^, we have found that this can increase the rate of background mutations, which would complicate an already difficult analysis. Because mutations will arise randomly during long term cell culture, we attempted at least three independent selections for each drug, in each case starting with an identical parental isolate (Fig. 1C). In a few cases, independent selections could not be achieved and dependent clones with a shared lineage (DOX-R4a and DOX-R4b; PTX-R2a and PTX-R2b; TPT-R4a, TPT-R4b and TPT-R4c) were collected. Resistance emerged after several months depending on the drug and the method used (7–30 weeks approximately, 49–210 generations) (Fig. S1).

Once resistance was observed in the batch culture, we isolated clones from batch drug-selected cultures and the drug sensitivity of the clone was measured and compared to the isogenic parent clones (Fig. 1D). We observed an EC_50_ fold shift between 3.3 and 411.7 (Table S2) in paired comparisons. To demonstrate that the drug resistance phenotype was stable, drug pressure was removed for 8 weeks (approximately 56 generations) and clones were retested for sensitivity. We observed no changes in the EC_50_ values, indicating that resistance phenotypes were not due to transient adaptation.

### Identification of putative resistance variants using next-generation sequencing

We next performed whole genome and exome paired-end read sequencing on the 35 cell lines (both drug-resistant clones and their matched drug-sensitive parent clones). Our comprehensive IVIEWGA studies in *Plasmodium*^5^, have shown that stable drug resistance is most frequently conferred by SNVs in coding regions and thus exome sequencing seemed initially the most cost-effective mechanism to find causal variants. However, gene amplifications, which contribute to 1/3 of drug resistance events in *Plasmodium*^5^, are more accurately detected with WGS because exact chromosomal recombination sites, which may fall in intergenic regions, can be reconstructed from WGS data. Because of falling costs over the course of the project, more samples (N = 21) were ultimately whole genome sequenced than whole exome sequenced (N = 14).

Sequencing quality was high for all samples: alignment showed that, on average, 99.9% of 700 million WGS (40 million WES) reads mapped to the hg19 reference genome with 86% of the bases covered by 20 or more reads (Table S3). By comparing sequences of evolved clones to their respective parental clones, we discovered a total of 41,259 SNVs (Table S4), of which 26,625 were unique (Table S5, “Methods”). The majority of variants in all cell lines were non-coding (Tables S4, S5) and were evenly distributed with respect to chromosome length (Fig. S2). Of the 26,625 mutations almost all (26,468) were present at allele frequencies (AF) of less than 85% relative to their parent clone and would thus not be expected to be driver mutations, given that the parents were cloned (to the best of our ability) before selections were initiated. The five drugs varied in the number of mutations, with TPT having the highest overall numbers (Table 1).

**Table 1:** Summary of average number of mutations.

	WGS				WES				
	DOX (n = 3)	GEM (n = 3)	PTX (n = 4)	TPT (n = 3)	TPT (n = 3)	DOX (n = 3)	ETP (n = 3)	GEM (n = 3)	PTX (n = 3)
Indels									
Disruptive inframe ins	0.00	0.00	0.00	0.00	1.00	1.00	0.00	0.00	0.00
Frameshift	0.00	1.00	1.33	2.33	0.00	1.00	1.00	1.00	1.00
Frameshift plus stop-gained	0.00	0.00	0.00	0.33	0.00	0.00	1.00	0.00	0.00
Inframe insertion	0.00	0.00	1.00	0.33	0.00	0.00	1.00	0.00	0.00
Intergenic	27.67	43.00	26.75	24.67	47.67	2.00	3.50	1.00	1.00
Intragenic	10.00	5.67	9.00	9.00	14.33	1.00	2.00	1.00	1.00
Intron	12.00	20.33	16.25	15.67	32.33	1.00	1.00	4.00	1.50
Splice region plus intron	0.00	0.00	0.00	103.3	0.00	0.00	0.00	1.00	0.00
SNVs									
Disruptive inframe del	0.00	1.00	1.00	0.67	0.00	1.00	0.00	1.00	1.33
Frameshift	1.00	2.00	1.00	22.33	2.00	3.00	1.67	1.00	3.00
Inframe deletion	0.00	0.00	1.00	0.00	0.00	0.00	2.00	0.00	0.00
Intergenic	898.33	1303.3	1416.6	258.67	2635.7	25.00	19.00	17.00	21.33
Intragenic	272.00	403.33	389.25	77.67	834.67	15.33	12.33	10.33	11.33
Intron	448.67	701.33	764.00	128.3	1358.33	28.00	27.00	33.00	22.67
Missense	16.00	14.00	12.75	7.00	34.33	15.00	19.67	21.67	15.67
Others	1.00	1.00	0.00	0.00	1.33	1.00	1.00	1.50	1.00
Splice region plus intron	1.00	1.67	2.00	30.00	3.33	1.67	1.50	1.00	1.33
Start lost	0.00	0.00	0.00	0.00	0.00	0.00	0.00	1.00	1.00
Stop gained	0.00	2.50	1.00	0.00	1.67	1.50	2.00	2.67	0.00
Stop lost	0.00	1.00	0.00	0.00	0.00	0.00	0.00	0.00	0.00
Synonymous	3.00	7.33	4.00	6.33	7.67	6.67	5.33	7.67	5.33

Number of selections performed with the drug is given by n. SNVs and Indels were grouped according to snpEff sequence ontology annotations (“Methods”, Table S9), and detailed counts per clone can be found in Table S4.

We next developed a pipeline (Fig. S3A, “Methods”) to filter the 26,625 “called” mutations (Table S5) to a final list of potential variants conferring drug resistance (Table S6). Our previous analyses in other species suggested that variants presented in coding regions are more likely to contribute to drug resistance even though this could exclude the variants associated with certain transcription factor (TF) binding sites. Therefore, our strategy focused on mutations that were in exonic regions and were drug-specific (Fig. S3A). We further considered only mutations likely to have a functional impact at the protein level (missense, nonsense, frameshift, start or stop gain or loss) which further reduced the number to an average of 35 and 23 nonsynonymous mutations for WGS and WES, respectively (Fig. S3A). Reasoning that resistance driver mutations (e.g. those actually causing resistance) would be present in 100% of the haploid cells in the sequenced culture, we selected only the variants with high allele frequency (AF > 0.85, as determined by sequencing read count). The top 2.5% of highest AF mutations corresponded to an AF > 0.85 (Fig. S3B). At this cutoff, the majority of cell lines harbored a candidate resistance mutation. While selecting a cutoff represents a tradeoff with potentially missed relevant mutations, the full list of mutations is provided in the supplement (Table S5). We did not note any strong correlation between read depth and allelic fraction in our study (R2 = 0.06; Fig. S3C) and all of the final mutations selected for further analysis had a read depth > 10 reads, with the majority supported by over 20 (Fig. S3D). Although some with AF < 0.85 could confer a beneficial advantage to the cell, most are likely to be random mutations that arose during long term culture. Finally, our experience with microbes has shown that genes which are not from gene families that are known to be excessively variable and which show multiple, independently derived amino acid changes will have high rates of confirmation using independent methods. Considering this allowed us to add in 4 genes (*STARD9, CYP1B1, SLCO3A1* and *DCK*) to our final list of 21 candidates (Table S6) despite these having slightly lower AF numbers (e.g. *DCK* AFs were 0.78 or less). We also identified a group of 298 genes with lower AF values that were either recurrent with disruptive alleles or were found in CNVs (Table S7).

Although they may theoretically play a role in resistance, we did not consider intergenic mutations nor intron variants because their high numbers suggested they could more likely arise by chance. Altogether only 465 (~ 1%) of the 41,259 total SNVs were predicted missense mutations in our complete dataset (Tables S4, S5) while 11,613 and 19,127 variants encoded a non-splice site intronic variant (underestimation due to WES samples) or intergenic mutation, respectively. Despite this, we did find several intronic mutations in genes that have a high probability of conferring resistance, including in SLCO31A (3) and TOP1 (1). We found two intragenic mutations upstream of DCK but one was in an unexpected line (PTX not GEM) and both were more than 100,000 kb away from DCK.

### Somatic copy number variations (CNVs)

We next searched for CNVs (both amplifications and deletions) in our WGS and WES data using Control-FreeC^32^. Overall patterns of broad and focal alterations across the drugs and conditions varied (Fig. S4A, Table S8). Using a corrected p-value of less than 0.05, we identified 93 total amplification and 108 deletion events, with most appearing in the TPT-resistant samples (123) (Table S8). The CNVs had an average size of 8.5 Mbp (stdev 19 Mbp), ranged from 15,000 bp to 152 Mbp (Fig. S4A) and covered ~ 3% of the genome, on average. More CNVs were called in WES samples because of sequencing gaps—even for WGS samples, some CNVs were separated by short distances and were nearly contiguous (Fig. S4A). It is likely that some CNVs were also missed in the WES data. The number of events was proportional to chromosome size, with the exception of the Y chromosome, for which there were ~ 4 × more events (47) per unit length. Some CNV calls were supported by paired end red data, for example, the one near WWOX (Fig. S4B,C).

### Doxorubicin resistance is associated with mutations in *TOP2A* and a solute carrier transporter

**Figure 2:**
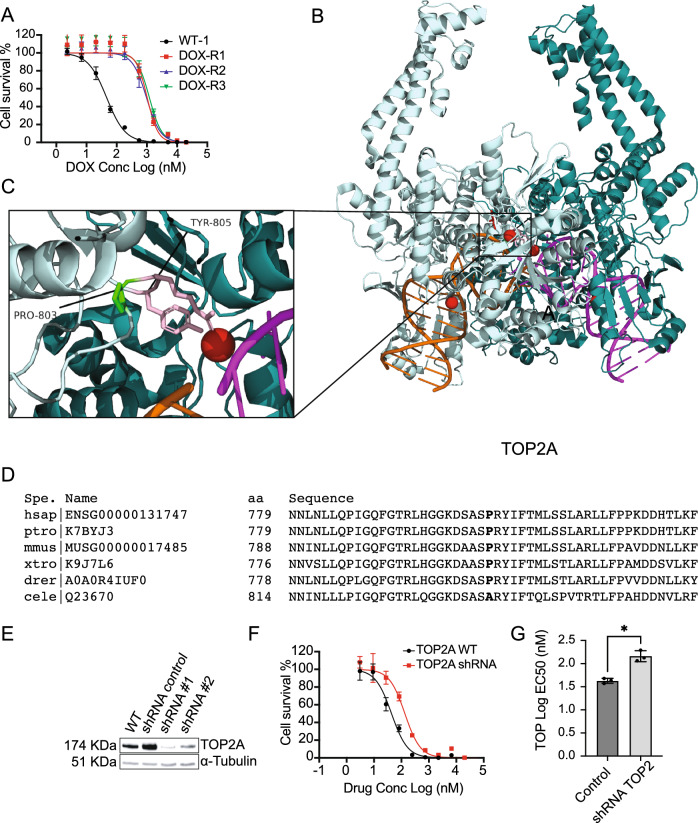
(**A**) DOX EC_50_ curves for DOX evolved clones using 8 technical replicates for each concentration (**B**) Crystal structure of human TOP2A dimer (subunits shown in teal and pale cyan) in complex with DNA (4FM9) and inset (**C**) showing location of Pro803 (green) in relationship to catalytic arginine and tyrosine (804, 805, pink) and Mg2 + (red). (**D**) Alignments of the TOP2A sequence subsets showing conservation of the Pro803 residue (bold) in all vertebrates but not *Caenorhabditis elegans* (cele). (**E**) Western blot confirming that shRNA gene depletion downregulates TOP2A protein level. shRNA #1 and shRNA #2 indicate independent biological replicates with same pool. (**F**) EC_50_ curves of the WT and shRNA (shRNA #1) knockdown cell lines for *TOP2A*. (**G**) Barplot of the WT and shRNA knockdown cell line (shRNA#1) for *TOP2A*. *p value < 0.05. P values determined by a paired ratio *t* test with EC_50_s determined by three independent biological replicates.

To evaluate the approach, we next considered the set of SNVs and CNVs for each drug. For DOX, we had six available selections from two different starting clones (WT-1 (WGS) and WT-5 (WES)) that were analyzed by WGS (DOX-R1, DOX-R2, DOX-R3 (Fig. 2A)) and by WES (DOX-R4a, DOX-R4b and DOX-R5). High allele frequency missense mutations were found in only 11 genes (Table S5). Of note, DOX-R2 and DOX-R3 carried mutations in *TOP2A* at frequencies of 0.89 and 0.87, respectively. *TOP2A* is the known target of DOX^21,33^ and is known to play a role in drug resistance^33–35^. The amino acid mutation, Pro803Thr (Fig. 2B,C), sits within the principal DNA-binding locus, the DNA-gate, a region conserved in type II topoisomerases (*TOP2A* and *TOP2B*). It is also adjacent to the catalytic tyrosine (Tyr805), responsible for nucleophilic attack on DNA (Fig. 2B,C)^36^. The Pro803 site is highly conserved in metazoans as is *TOP2A* generally (Fig. 2D). Examination of the human *TOP2A* DNA-bound crystal structure suggests that Pro803Thr is a gain of function mutation that may block DOX-intercalated DNA from binding, thus preventing the *TOP2A*/DNA/DOX adduct from poisoning the cell. Alternatively, it could be a loss of function mutation, especially as knockdown of *TOP2A* activity has previously been shown to confer DOX resistance in a Eμ-Myc mouse lymphoma system^37^. Downregulation of *TOP2A* using a pool of target-specific shRNA hairpins (confirmed by Westerns, Fig. 2E) resulted in a modest resistance (~ 4X, Table 2) that was not equivalent to the resistance seen in the evolved cell lines (Fig. 2F,G). Although these knockdown results recapitulated those of others, we favor the hypothesis that the mutation is a gain-of-function allele. Indeed, others have identified and validated the TOP2A Pro803 residue as important for drug resistance using recombinant protein expression and a yeast model^38^.

**Table 2:** Validation (knockdown) results for selected genes.

Drug	Sample	Gene	Amino acid change	Type	AF	EC_50_ (nM) WT	EC_50_ (nM) KD/KO/CI
DOX	R1, R2, R3	TOP2A	Pro803Thr	MS	0.89, 0.87	38.6 ± 4.3	164.3 ± 43.9
	R4b	SLC13A4	Gly165His	MS	1	52.9 ± 11.6	204.3 ± 35.7
	R5	SPG7	Lys593Asn	MS	1	NE*	
PTX	R1, R2a, R3	WWOX	16q23.1	CNV	–	5.8 ± 2.5	42.9 ± 11.7
	R2a, R2b	ABCB1	7q21.12	CNV	–	252 ± 38 218 ± 14	1.3 ± 0.1 1.3 ± 0.1
	R2b, R6	SLCO3A1	Ile587Asn (R2b), Ala263Thr (R6)	MS		6.6 ± 3.3	51.5 ± 9.9
GEM	R1, R2, R3	DCK	Ser129Tyr (R1, R2), Asn80fs (R1. R3); Asn113fs (R2); Thr184fs (R3)	MS, FS		14.3 ± 1.7	521.7 ± 58.3
	R4, R5, R6	RRM1	11p15.4	CNV	–	54.9 ± 5.8	1.8 ± 0.1
ETP	R2	WNT3A	1q42.13 (R2)	CNV	–	ND	–
	R3	WDR33	P622T	MS	1	241.5 ± 31.0	821.6 ± 226.9
TPT	R1, R4a, R4b, R4c	CYP1B1	Val432Leu; Asp217Glu (R4a,b,c)	MS	0.13, 0.40, 0.43, 0.42	6.3 ± 0.2	13.3 ± 0.3
	R1, R2, R3	WWOX	16q23.1	CNV	–	2.4 ± 0.3	22.8 ± 2.7
	R4a, R4b, R4c	TOP1	His81fs; 20q12	FS; CNV	1	ND	–
	R4a, R4b, R4c	USP47	Arg408*	Stop	0.38, 0.57, 0.58	3.05 ± 0.2	1.07 ± 0.07

CNV, copy-number variant. MS, missense. FS, frameshift variant, KO/KD/CI, knockout, knockdown, chemical inhibition (verapamil, ABCB1). ND: No data: gene knockdowns were attempted but could not be achieved. NE: Not expressed (protein not detected by Western blot, preventing validation). *EC*_*50*_* WT and EC*_*50*_* KO/KD/CI* are from matched pairs for the given drug and represent the mean ± s.e.m. (n = 3 biological replicates).

We also observed some lines bore missense mutations present in 100% of the reads for several other attractive but less well characterized genes; *SLC13A4* (Gln165His, DOX-R4b), and *SPG7* (Lys593Asn, DOX-R5, AF = 1), as well as one uncharacterized gene (AC091801.1, His13Asn, DOX-R4a) in the three different clones that were subjected to WES and which were derived from WT-5. *SLC13A4* is a solute carrier transport family member and members of this general solute carrier family have appeared in selections conducted in microbes (e.g. the UDP-galactose transporter and the AcetylCoA transporter^39^) and are also associated with cancer drug resistance^40^. The Gln165His mutation is located in a relatively disordered region of the protein making it less attractive as a candidate. *SPG7* (also called paraplegin) is a gene associated with spastic paraplegia in humans and would initially not seem to be a plausible drug resistance gene. It encodes one subunit of the mAAA (matrix ATPase associated with diverse cellular activities) protease, which is located in the mitochondrial matrix^41^. In particular, mutations to mitochondrial genes in *C. elegans* have been shown to confer resistance to hemiasterlin, an antimitotic agent that functions by disrupting microtubule dynamics^42^. Human variants have been shown to elevate mitochondrial reactive oxygen species^41^. shRNA knockout experiments were not successful with *SPG7* and it seems likely that CRISPR-Cas9 editing would be needed to confirm a predicted gain of function, but it remains an attractive candidate based on work in other species. *SPG7* is one of 170 genes included in a set of drug metabolizing enzymes and transporters, that also includes *DCK* (see below), *CYP1B1*, *SLCO3A1* and *ABCB1* and shows an association with resistance to docetaxel^43^ and as with *TOP2A*, the mutated region is highly conserved across vertebrates.

### Gemcitabine resistance is conferred by changes in DCK and RRM1 activity

**Figure 3:**
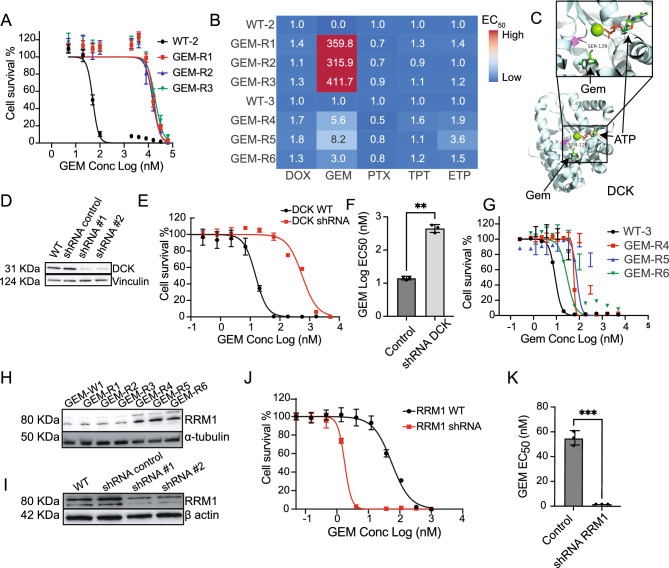
(**A**) GEM EC_50_ curves for first set of GEM evolved lines using n = 8 technical replicates per concentration point (**B**). EC_50_ ratio matrix showing absence of multidrug resistance pathways in all GEM resistant lines. (**C**) Crystal structure of human DCK (1P62) co-crystalized with GEM showing the position of Ser129, ~ 11 Å from GEM. D. Western blot confirming that shRNA gene depletion downregulates protein levels for *DCK*. shRNA #1 and shRNA #2 indicate independent biological replicates with same pool. (**E**) EC_50_ curves of the WT and shRNA knockdown cell lines for *DCK*. n = 8 with individual technical replicates overlaid for every concentration point. (**F**) Barplot of the EC_50_ WT and shRNA knockdown cell line for *DCK* in presence of GEM. Significance (**p < 0.05) determined by a paired ratio t test for three biological replicates. (**G**) GEM EC_50_ curves for second set of GEM evolved lines. (**H**) Western blot for *RRM1* across all GEM samples showing overexpression pattern of *RRM1* in GEM-R4-6 resistant clones. *γ-tubulin* is used as a loading control. I. Western blot confirming that shRNA gene depletion downregulates protein levels for *RRM1*. shRNA #1 and shRNA #2 indicate independent biological replicates with same pool. *β-actin* is used as a loading control. (**I**) EC_50_ curves of the WT and shRNA knockdown cell lines for *RRM1*. (**J**) Barplot of the EC_50_ control and shRNA *RRM1* knockdowns in presence of GEM. Significance (***p < 0.01) determined by a paired ratio t test for three biological replicates.

Six selections were performed with GEM (starting from two different isogenic parents; WT-2 (WGS) and WT-3 (WES)). Among those, three GEM-resistant clones subjected to WGS (GEM-R1, GEM-R2 and GEM-R3) showed an average EC_50_ shift of 300 to 400-fold (Fig. 3A, Table S2), and the clones showed no change in HAP1 sensitivity to other drugs (Fig. 3B). As there were no candidate alleles with AF > 0.85, we looked for genes that acquired mutations in multiple selections, identifying deoxycytidine kinase (*DCK*) as likely important for resistance. Interestingly, across cell lines several distinct mutations were found in *DCK*, with varying effects (missense and frameshift) across several different positions (Table 2). In particular, the missense substitution, Ser129Tyr, present in GEM-R1 and GEM-R3, not only alters the amino-acid size and charge but also falls within the known GEM binding pocket in a DCK/GEM crystal structure (1P62^44^), making it an exceptionally strong causal candidate for GEM drug resistance (Fig. 3C). GEM is a prodrug that only becomes pharmacologically active if it is phosphorylated by DCK^44^. A shRNA knockdown of DCK was performed and confirmed by western blot analysis (Fig. 3D). Although the mutation appears to be a gain of function allele, downregulation of the DCK nevertheless resulted in a 36.5-fold increase in the EC_50_ value compared to both the isogenic parent line and the shRNA negative control (Fig. 3E,F; Table 2).

The three WT-3 derived GEM-resistant clones (GEM-R4, GEM-R5 and GEM-R6) subjected to WES were not as resistant as those used in WGS (~ 6× versus ~ 400×, Fig. 3G, Table S2). Our work in other species with well characterized compounds suggests this is not surprising and that even single nucleotide changes in the same gene can yield different levels of resistance. For example, repeated selections with dihydroorotate dehydrogenase (DHOD) inhibitors in a mouse model and in vitro culture gave rise to 13 different point mutations in parasite DHODH with levels of resistance ranging from 2- to ~ 400-fold^45^. No high AF SNVs were evident in these lines and *DCK* exons were not mutated. On the other hand, the three WES clones contained 20 CNVs that could play a role in drug resistance. Most CNVs were not shared between lines but GEM-R4, GEM-R5 and GEM-R6 all bore overlapping CNVs of varying sizes on chromosome 11, with all three lines bearing 3–4 copies (p value = 1.38e − 37 to 2.05e − 142) (Fig. S4). The chromosome 11 CNV was only found in GEM resistant lines and not in any of the other evolved lines (in contrast to CNVs on chromosome 1 or 16, for example). While it is difficult to determine which of the 140 genes in the smallest interval contribute to resistance, a known resistance mediator or target of GEM, ribonucleotide reductase (*RRM1*), was found within the amplified region. RRM1 is the largest catalytic subunit of ribonucleotide reductase, a key enzyme catalyzing the transformation of ribonucleotide diphosphates to deoxyribonucleoside diphosphates that are required for DNA synthesis and repair, and GEM is known to inhibit DNA polymerase by inhibiting *RRM1*^46^. Furthermore, overexpression of RRM1 is associated with poorer prognosis after gemcitabine treatment in non-small cell lung cancer^47^ and in bladder cancer^48^.

Western blot analysis of the evolved lines showed that the amplification was indeed associated with increased protein levels (Fig. 3H). As an additional validation, we performed shRNA knockdown of *RRM1* to reduce protein expression (Fig. 3I), followed by a dose–response assay comparing EC_50_ values of both wildtype HAP1 and *RRM1* knockdown lines, which showed that downregulation of *RRM1* made HAP1 cells 31-fold more sensitive to GEM than their isogenic parent (Fig. 3J,K). As expected *RRM1* downregulation had no effect on HAP1 sensitivity to other drugs (Fig. S5).

### Paclitaxel resistance is mediated by transporters *SLCO3A1 and ABCB1*

**Figure 4:**
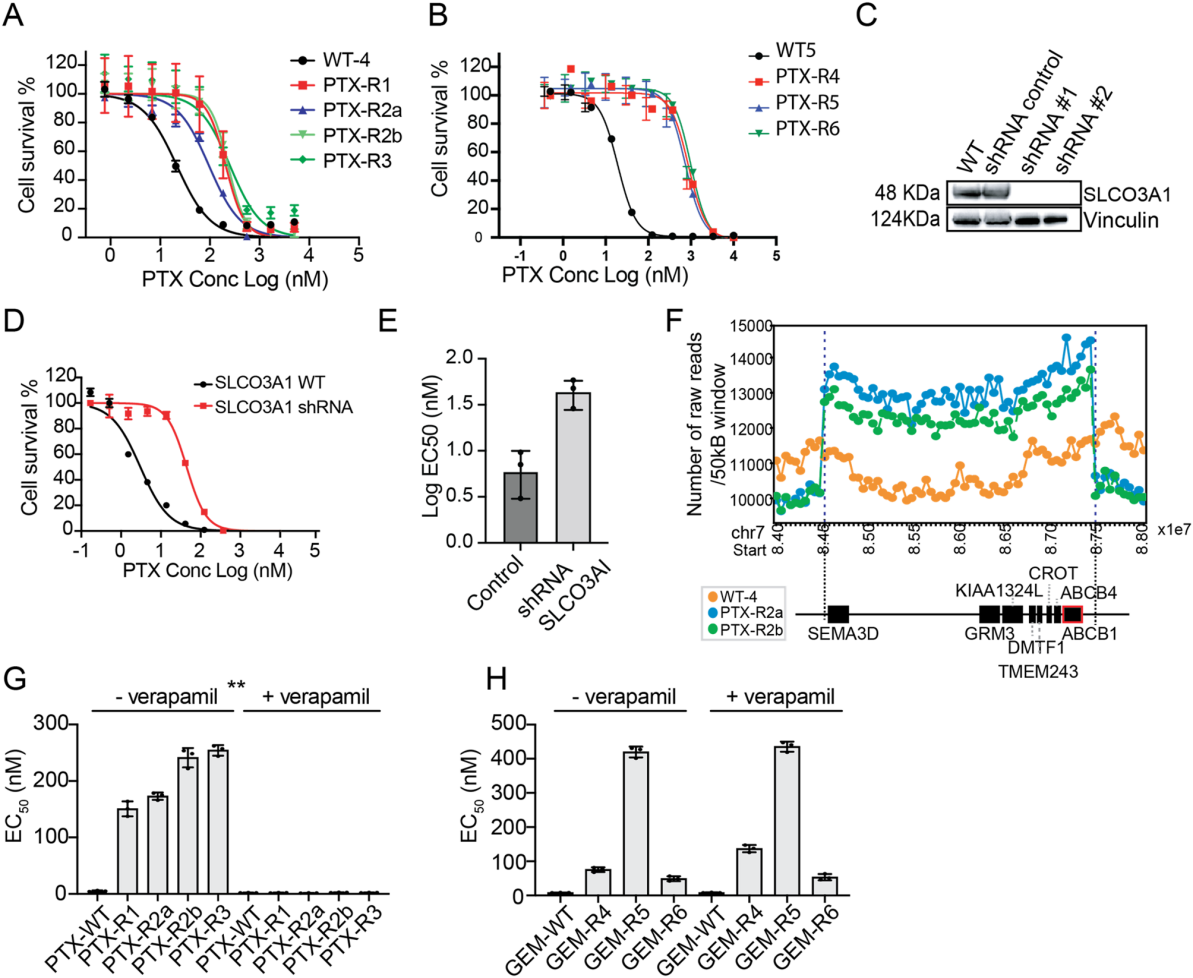
(**A**) PTX EC_50_ curves for evolved lines with n = 8 technical replicates (values are in Table S2) (**B**) PTX EC_50_ curves for a second independent set of evolved lines with n = 8 technical replicates (**C**) Western blot confirming shRNAs downregulate protein levels for *SLCO3A*. shRNA #1 and shRNA #2 indicate independent biological replicates with same pool. (**D**) EC_50_ curves of the WT and shRNA knock-down cell lines. (**E**) Barplot of the WT and shRNA knockdown cell lines for *SLCO3A1* for three independent biological replicates. (**F**) Raw copy number profile for the amplification event containing protein coding genes including ABC transporters (*ABCB1/ABCB4)* PTX cell lines. The amplification region (chr7:84500000-87300000) had a higher number of raw reads (labeled with blue dash lines) with default window size of 50 K bp. Genes associated with the CNV event are depicted by black boxes underneath according to their position and sizes. *ABCB1* is highlighted with red outline. (**G**) Barplot of EC_50_ of the PTX treated cell lines with and without verapamil and verapamil alone showing sensitization in presence of verapamil an ABC inhibitor (n = 4 technical replicates). (**H**) Control barplot of EC_50_ of the GEM cell lines ± verapamil showing no EC_50_ shift for GEM cell lines when co-treated with verapamil. Unless otherwise noted, all data is represented by mean ± s.e.m. with n = 3 with individual biological replicates overlaid. **p value < 0.01. p values determined by two-tailed *t* test.

Seven different paclitaxel lines were created with different resistance levels (PTX-R1, R2a, R2b and R3, ~ 10× to PTX-R4, R5, R6, 50X) (Table S2). The first four (Fig. 4A) were subjected to WGS and the latter three to WES (Fig. 4B). SNV analysis yielded no candidate genes (frameshift, indels, and missense mutations with an allele frequency > 0.85). From genes with an allele frequency of less than 0.85, *SLCO3A1,* encoding another solute carrier transporter, was notable in that multiple missense alleles were identified (Ile587Asn, Ala263Thr). This class of transporter is known to play a role in the import of drugs as well as hormones such as prostaglandin^49^. Gene knockdown experiments showed that clones with loss of SLCO3A1 (Fig. 4C) resulted in HAP1 cells that were ~ 8 times more resistant than their isogenic parents to PTX (Fig. 4D,E).

Despite the lack of obvious coding SNVs, PTX-R1, R2a, R2b and R3 had a combined number of 47 CNVs, while PTX-R4, R5 and R6 had 10 (the fact that more CNVs were found in WGS samples may reflect the ease with which CNVs are called with WGS versus WES data). Potentially significant genes with CNVs were *ABCB1* (MDR1) and *ABCB4* (*MDR3*) (Fig. 4F) on chromosome 7 (PTX-R2a, R2b). *ABCB1* amplifications are associated with clinical resistance to PTX^50^. PTX-R4 and R5 showed structural variants on chromosome 1, and PTX-R4 show an amplification event on chromosome 17 that encompassed a variety of ABC transporters (*ABCA5, 6, 8, 9, 10*). No compelling candidate genes were found in CNVs for PTX-R6. On the other hand, inspection with IGV showed that read coverage was poor and that CNVs might not have been detected with WES data.

To confirm the importance of ABC transporters in PTX resistance, clones were treated with both PTX and verapamil, a calcium channel-blocker which can reverse ABC-transporter mediated resistance^51,52^. We observed a complete reversal of resistance in PTX lines (Fig. 4G). In contrast, we observed no reversal of resistance in GEM lines (Fig. 4H), suggesting the resistance role of ABC-transporters is PTX-specific.

### Etoposide resistance is modulated by levels of *WDR33*

We created three independent ETP resistant clones, all of which were subjected to WES, and compared them to one isogenic parent clone (WT-3) (23, 13 and ninefold increased resistance respectively (Fig. S6A, Table S2). A single gene, *WDR33* (ETP-R3), carried a SNV (Pro622Thr) with a 100% allele frequency. This gene encodes for a member of the *WD* repeat protein family and is one of the six subunits of a multiprotein complex called CPSF (cleavage and polyadenylation specific factor)^53^ involved in cell cycle progression, signal transduction, apoptosis and gene regulation. Members of the CPSF complex have appeared repeatedly in in vitro evolution experiments in parasites (both in *Toxoplasma gondii* and *P. falciparum*) and have been validated as resistance mechanisms^54,55^. While CPSF subunits may represent targets of specific drugs, point mutations in members of the complex may also have a role in overcoming cellular stress that blocks cellular proliferation, Disruption of *WDR33* can lead to slowed DNA replication forks^56^, which could potentially explain why its disruption protects against topoisomerase inhibitors that block DNA unwinding. Lines in which *WDR33* was knocked down via shRNA acquired an EC_50_ value 3.4 times greater than its parental line or the scrambled control (Fig. S6B–D; Table 2), despite an incomplete disruption of the gene by shRNA silencing. ETP-R3 also has a Cys56Phe WNT3A mutation that is found in a highly conserved cysteine that participates in disulfide bond formation and whose disruption abolishes WNT3A activity in recombinant assays^57^. WNT3A has been linked to Etoposide resistance as well as resistance to multiple chemotherapies^58^ (reviewed in Ref.^59^).

No clear candidate SNVs were evident for ETP-R1 and ETP-R2, which did not carry the *WDR33* or *WNT3A* mutations (Table S5). We also noted that all the three ETP resistant lines were cross-resistant to PTX, and DOX, slightly cross-resistant with TPT (Fig. S6E,F) but showed no sensitivity to GEM (Fig. S6G). Despite the cross-resistance, we did not observe any overrepresented genes with missense mutations or shared CNVs that were found in the ETP, PTX and DOX lines but not GEM (Table S7). Because automated analysis of WES could miss smaller CNVs and because *ABCB1* has been linked to both ETP, DOX and PTX resistance ^60^ but not GEM, we examined the *ABCB1* region manually using IGV. These data showed a large increase in read coverage for EPT-R1 and EPT-R2 relative to WT-3 parent line (Fig. S6H) at the same chromosome 7 *ABCB1* region amplified in the PTX lines (Fig. 4F). As a final step, we tested PTX lines (PTX-R1, R2a, R2b and R3) that contain amplifications of *ABCB1*, and they were indeed cross-resistant to ETP (Fig. S6I). These data show the limitations of WES data and current automated algorithms. Deciphering the contributions of amplified genes in the absence of a rich literature may require other technologies such as genome-wide knockdown or overexpression libraries.

### Topotecan resistance is associated with complex alterations in *TOP1*, deletion of *WWOX* and SNVs in cytochrome p450s (*CYP1B1*)

The six TPT samples were derived from four independent selection events (TPT-R4a-c are clones from the same selection with levels of resistance ranging from 10 to 20×; Table S2) and all six clones were subjected to WGS together with their parent clones (WT-6 and WT-7).

**Figure 5:**
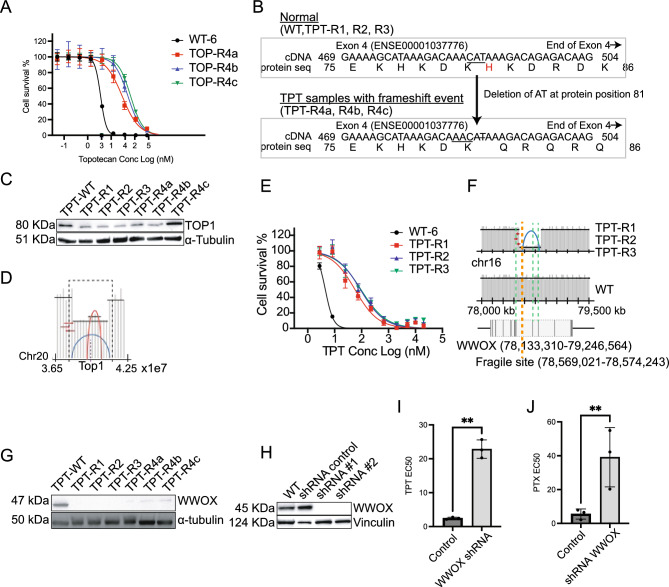
(**A**) TPT EC_50_ curves for TOP-R4a,b and c. (**B**) Transcript sequence (cDNA) and protein sequence for TOP1 transcript for normal (top) and TPT 4a, b and c. The figure shows only part of the cDNA (position 469–504) and protein sequence (position 75–86) for TOP1 at the affected exon (Exon 4, ENSE00001037776). The frameshift deletion of nucleotides ‘CAT’ to ‘C’ observed in TPT samples (TPT-R4a, R4b, and R4c) is predicted to give a frameshift at amino acid 81 (His, red highlight in normal). Amino acids affected by the frameshift deletion are highlighted in red. (**C**) Western blot TOP1 protein depletion in evolved lines*.* (**D**) Schematic showing complex read depth patterns around *TOP1*. (**E**) TPT EC_50_ curves for evolved TPT-R1, R2 and R3. (**F**) Schematic of chr16 reads around *WWOX* for TPT-R1, R2, and R3 compared to the WT chromosome 16 parental cell line. Blue arch represents a deleted region. *WWOX* below shows the exonic (black lines) and intronic (white box) regions of the gene. The start of the deletion event is also close to a known fragile site (orange dashed line). (**G**) Western blot showing WWOX protein levels in TPT resistant clones. (**H**) Western blot shows downregulation of protein levels for *WWOX* in shRNA samples compared to WT and scrambled control. shRNA #1 and shRNA #2 indicate independent biological replicates with same pool. Barplot of EC_50_ control (wt) and WWOX shRNA knockdowns for TPT (**I**) and PTX (**J**). EC_50_ data is represented by mean ± s.e.m. with n = 3 biological replicates and 4–8 technical replicates. ** = p < 0.05. p values determined by ratio, paired two-tailed *t* test.

For TPT-R4a-c lines (Fig. 5A), 268 alleles were present with AF > 0.85, but of these, only six were coding mutations and the rest were intergenic. Three of the six coding mutations were frameshift mutations (His81) with AF = 1 in *TOP1* (Fig. 5B, Fig. S7A), the known target of topotecan^23^. The His81 frameshift mutation, which introduces a premature stop codon, was confirmed by examining the read alignments (Fig. S7A) and by the absence of the full-length protein using N-terminal antibodies (Fig. 5C). Because there were also complex structural variants in the region (Fig. 5D, Fig. S7B) we also sequenced the 5ʹ cDNA through the His81 frameshift for all three lines and as well as the parent line and confirmed the two-base deletion in the mutant as well as homozygosity in TPT-R4a-c evolved lines. We also observed a decrease in mRNA expression with TPT-R4a-b showing a statistically significant decrease in TOP1 mRNA expression, relative to TPT-WT (Fig. S8). It has been previously shown that a targeted RNAi suppression of Top1 produces resistance to camptothecin, a close analog of topotecan^37^. Interestingly, of the 22 TOP1 frameshift or nonsense mutations in the COSMIC tumor database, 6 were located within a 30 amino acid span (of 765 total) that includes His81 (exon 4), suggesting likely clinical relevance^61^. The probability of this distribution by chance is 9.65 × 10^−5^.

No clear coding SNVs with a high allele frequency were obvious in TPT-R1, R2 and R3 (Fig. 5E) but we noted multiple SNVs (Asp217Glu from TPT-R4a, b, c and Val432Leu from TPT-R1) in *CYP1B1*, which encodes a cytochrome p450 isoform. Overexpression of CYP1B1 has previously been associated with TPT resistance^51^. TPT resistant lines (TPT-R1, R2 and R3 also showed large chromosomal abnormalities at *WWOX* (Fig. 5F) with a clear deletion of the *WWOX* gene region (chr16:78569166–78792736, exon7 and 8). WWOX bears a well-known fragile site (FRA16D) and encodes a putative oxidoreductase. The complete absence of WWOX protein was confirmed by Western in TPT-R1, 2 and 3 (Fig. 5G). Interestingly, lower levels of WWOX were also observed in TPT-R4a-c, which could be a consequence of other *cis* or *trans* variants in this cell line and might also contribute to this level of resistance. Knockdown of *WWOX* by shRNA resulted in marked resistance to TPT (Fig. 5H,I). WWOX acts as a tumor suppressor and plays a role in apoptosis. Its disruption may prevent TPT-induced apoptosis, promoting cell survival in the presence of TPT^62^. WWOX disruption also resulted in resistance to PTX (Fig. 5J), and as reported by others who examined WWOX-transfected epithelial ovarian cancer cells^63^.

## Discussion and conclusions

Here, we show for the first time that in vitro evolution and whole genome analysis (IVIEWGA) can readily lead to the identification of drug resistance mechanisms in human cells. This method is designed to identify the targets of drugs that block cellular proliferation and could be used on any compound that has an antiproliferative effect, including chemotherapies such as asparaginase. The method could also theoretically be used with biologics such as a monoclonal antibodies or antisense oligonucleotides. In drug development, it could be used to demonstrate on-target activity. It would not be as useful on compounds that only change cellular signaling or those that alter the immune function, like prednisolone. Our results show in vitro resistance acquisition and provide a framework for the determination of chemotherapy resistance alleles that may arise in patients.

Our work using IVIEWGA in pathogens (see Ref.^64^ for a review) guided our pipeline development: We focused on protein coding alterations that arose in association with a single treatment condition, that were nonsynonymous, occurring repeatedly and were high allele frequency. We also removed alleles for genes that are known to mutate frequently, like odorant receptors. Overall, our results are similar to what we have observed in eukaryotic pathogens with a mix of CNVs and single nucleotide variants giving rise to resistance.

Because of the substantially greater costs associated with WGS, here we did evaluate both WES and WGS sequencing methods. Despite a higher likelihood of discovering all changes by WGS, the disadvantage of WGS is cost and computational time. While human WES data can be analyzed on a laptop, human WGS data files are large and difficult to handle, computationally. One potential strategy is to perform low pass WGS for CNV detection and WES for SNV detection on all samples.

The biggest disadvantage of using WES is that CNVs will be harder to call. This is partly for statistical reasons with many reads that support CNV calls located outside of coding regions in WGS samples. In addition, if one sequences over the exact location of the recombination event (or the start or end of the deletion) one can obtain additional support for location calls via split read analysis of paired-end libraries. In addition, one can extract the sequence of the short read and reconstruct the exact recombination breakpoint, as shown in Fig. S3. This would not be feasible with whole exome sequencing. Recently it was shown that CNV detection tools perform poorly on WES cancer genome samples. Comparative analysis showed a low consensus in CNV calling tools with moderate sensitivity (~ 50% to ~ 80%), fair specificity (~ 70% to ~ 94%) and poor FDRs (~ 27% to ~ 60%). Also, using simulated data these authors observed that increasing the coverage more than 10× in exonic regions did not improve the detection power^65^. Of course, detecting CNVs is likely to be more challenging in diploid genomes, than haploid genomes. In support of this, we were able to identify and validate the RRM1 amplification event in GEM-R4, 5 and 6, which were only subjected to WES. In addition, in yeast, it appears CNVs are much less important than SNVs in driving drug resistance as well: in a more comprehensive in vitro evolution study in yeast^66^ with 80 different and 355 whole genome sequences we observed only 24 CNVs, including apparent aneuploidy (11 times, occurring in 10 clones) and small, intrachromosomal amplifications (13 times, occurring in 13 clones) in our set of 355 whole genome sequences ^67^.

A lesser disadvantage of WES is the rare possibility that resistance is conferred by an intergenic mutation, which would be missed by WES data. GWAS studies have frequently identified noncoding variants as important for phenotypes in the human population. It is likely that most of the mutations that are evolved here would have a negative impact on fitness and would not be tolerated in the germline and thus would not appear in human GWAS studies^68^. Our work in other organisms has shown that almost all resistance conferring SNVs or small indels are nonsynonymous changes that would be detected by both WES and WGS. In our comprehensive yeast study^66^, 271 mutations of the 1405 detected mutations in the 355 evolved lines were intergenic. Of these, only five were directly upstream or downstream of one of the 137 genes that were repeatedly identified in the study. In contrast to coding mutations, most intergenic mutations lacked any statistical support suggesting relevance and were likely to be background mutations^67^. Despite the lower probability that intergenic or other noncoding mutations may have functional effect, we recognize that there are examples from the literature where intergenic mutations have contributed to drug resistance. Non-coding RNAs such as *EGRF-AS1* and activating cis elements such as enhancers have previously been implicated in evasion of drug response^69–71^. The intergenic mutations with high allele frequency are present in our provided datasets and provide opportunity for reanalysis or for querying by those interested in a specific noncoding RNA or enhancer. It is feasible that even synonymous mutations could confer resistance if they altered the rate of protein folding. In addition, humans clearly have much more complex splicing patterns than microbes and indeed we found evidence for multiple intronic (and intergenic mutations) in genes with higher-than-expected rates of disruptive mutations and some level of validation (e.g. *SLCO31A, TOP1, DCK*) contributing to resistance in our dataset.

A limitation of our HAP1 study, as presented, and in contrast to our work in other species, is that despite some level of repetition, we seldom achieved strong statistical confidence by just performing selections and sequencing. This may not be unexpected. Evolution is, unfortunately, a relatively stochastic process even when working with the exact same starting clone. In the yeast study^66^ we only obtained the same allelic change in the same critical drug resistance gene a few times despite > 3 repetitions per each of the 80 compounds. For example, two independent selections with hectochlorin both yielded an Arg116Lys in ACT1, the target of hectochlorin^67^. Similarly, a Leu671Phe change in YRM1 was observed 5 times for 4 different compounds.

Another disadvantage of using human cells is the challenge of validation of SNVs; we were not able to engineer any SNVs into HAP1 cells to demonstrate their importance. On the other hand, with the statistical confidence that comes from identifying the same gene repeatedly, CRISPR-*Cas9* validation becomes less important. In the same yeast study described above, YRM1, a gene encoding a transcription factor involved in drug resistance in yeast was independently identified 52 times with 27 different alleles^66^. The likelihood of 355 selections yielding the same gene by chance is roughly 3.53 × 10^−116^. This enrichment analysis becomes an attractive method for teasing apart driver and passenger mutations and may become possible with more repetitions despite the larger genome size of HAP1 cells. However, performing enough repetitions to achieve statistical confidence would require substantial resources with WGS, even with a thousand-dollar human genome. WES is thus likely to be more useful.

While HAP1 cells may not be considered a perfect model for human cancer biology, for the purposes of target identification, they are likely very useful. As with pathogens, our use of well-studied drugs, largely uncovered genes that were mostly already well known to confer resistance such as RRM1^46,72^, DCK^73,74^, TOP2A^37^. and TOP1^37^ in a variety of different cancer cell lines. Although it was initially argued that the in vitro evolution system might be artificial, in malaria parasites it has been used to discover or rediscover most, if not all (to our knowledge), clinically relevant drug resistance genes including the chloroquine resistance transporter^5^, the artemisinin resistance gene, *Pfkelch13*^75^, and well-known ABC transporters^5^.

Despite questions about how much they mimic human cells, the value of using haploid cell lines is evident from our allele frequency data. If our lines had been diploid, we would have needed to consider allele frequency data of up to 0.4. There are 205 missense mutations with an AF of > 0.4, making pinpointing the causative allele much more difficult without candidate genes or without many repetitions. Although in vitro evolution has been used repeatedly for discovering the mechanism of action of completely uncharacterized compounds in malaria parasites (reviewed in Ref.^64^), there are fewer examples of in vitro evolution being used for de novo target discovery in diploid eukaryotic pathogens. Although there are some examples in trypanosomes^76–78^, some hypothesis about the mechanism of action was already present before evolution studies were attempted. Despite this, low allele frequency data should not necessarily be discarded. There have been multiple examples from haploid Plasmodium where a resistance-conferring allele was located within an amplification region and thus showed an AF < 0.5. It is possible that the incomplete penetrance that we observed here with respect to AF is due to duplication events that were not readily detected. This may be particularly true for WNT3A (AF = 0.571) which lies in the chromosome 1 amplified region.

Although the HAP1 cells could be considered unnatural, it is likely that similar evolution experiments in other types of human cells will largely give the same genes. This is because conservation of drug targets and drug resistance mechanisms across phyla is often observed, although a given compound or inhibitor may show differences in selectivity and specificity. Resistance to topotecan/camptothecin in yeast is also provided by mutations in Top1^79^. Our IVIEWGA studies in yeast also identified Top1 as the target in yeast^66^. Evolution studies with cladosporin in yeast and plasmodium both give the same resistance mechanism for cladosporin, lysyl tRNA synthetase^80^.

Our studies were not meant to study the process of evolution. Within the field of laboratory-based evolution, there are two broad areas of study. The first are those that fall under the heading of “experimental evolution” and which try to mimic evolution in natural conditions. Here, growth rates are often recorded, and experimental conditions may be varied in a controlled manner (carbon sources, temperature, etc.). Such studies include long term studies of *E. coli* or other bacteria (reviewed in Ref.^81^) and have also been performed with small molecules^8,82,83^, primarily with known mechanism of action. Alternatively, there are also studies in which evolution has been used as a tool to discover targets and resistance genes for therapeutic purposes ^7,64,84^. In many cases^84,85^, although not in all cases the term “in vitro evolution” is used instead of “experimental evolution.” Based on our results here, resistance readily emerges in HAP1 cells but more work will need to be done to determine if this is because of the compounds that were used. Here we used in vitro evolution (versus experimental evolution) to select for mutant lines that could withstand treatment with the selected drugs. Although it may be possible to use HAP1 cells for experimental evolution, at present sequencing costs are so high that whole genome studies with whole genome analysis are not practical but this may change in the future. Questions that might be investigated include the fitness of different mutations, reproducibility of the process, impact of the starting clone, carbon sources or growth rate and whether one resistance mechanism predominates or if a variety are found.

Finally, it is important to keep in mind that the compounds examined here are not modern cancer therapies and while still used clinically, they are imperfect. Newer molecules include bortezomib, a small molecule proteasome inhibitor, imatinib, a small molecule tyrosine kinase inhibitor or seliciclib, small molecule cyclin-dependent kinase inhibitor or even small molecule cancer immunotherapies. A limitation of the cell line that was used here is that it is already resistant to some of these modern drugs, including imatinib (Fig. S1), which could necessitate the use/development of other haploid lines, or potentially the use of diploid lines, although data analysis is expected to be more difficult. We anticipate mutations in the drug’s targets will be identified sometimes, as is observed in microbes. In fact, unbiased IVIEWGA studies with bortezomib in *P. falciparum* have demonstrated mutations in the proteasome subunit, Pf20S β5^86^, confer resistance, and similar resistance-conferring mutations have been discovered after using in vitro evolution in human cells, although whole genome sequencing was not performed and the mutations were identified using a candidate gene approach^87^. On the other hand, some targeted therapies may not work well against HAP1 cells because they do not bear the sensitizing mutation (e.g. the *BRAF/EGFR* mutations for vemurafenib, gefitinib or erlotinib, respectively^88^). Alternatively, the HAP1 cells may be intrinsically resistant because they harbor other resistance conferring mutations, which may be the case for imatinib which targets the BCR-Abl fusion protein encoded by HAP1 cells. Nevertheless, if they do show sensitivity or can be engineered to sensitivity HAP1 cells may prove useful for predicting resistance mechanisms for new drugs in clinical development, for determining on-target activity and for studying the many other drugs that were not included in our small screening set.

## Materials and methods

### Compounds

All chemotherapeutic agents used in this study were obtained from Sigma-Aldrich, dissolved in DMSO at 10 mM concentration and stored at − 20 °C.

### Cell cultures

The human chronic myelogenous leukemia cell line, HAP1, was purchased as authenticated at passage 7 from Horizon Discovery and cultured in tissue culture dishes (Genesee Scientific, Cat# 25-202) as a monolayer at 37 °C in a humidified atmosphere with 5% CO_2_ using Iscove’s Modified Dulbecco’s Medium (IMDM) (Life Technologies, CA) supplemented with 10% fetal bovine serum (FBS), 0.29 mg/mL l-glutamine, 25 mM HEPES, 100U/mL Penicillin and 100 µg/mL Streptomycin (1% pencillin/streptomycin). Monoclonal and polyclonal stocks were made and stored in IMDM + 10% DMSO in liquid nitrogen.

### In vitro evolution of resistant HAP1 clones

Prior to selection, an aliquot of the parental line was stocked as a reference for subsequent whole genome sequencing analysis. Three independent clones of HAP1 cells were cultured in tissue culture dishes exposed to increasing sublethal concentrations of each chemotherapeutic agent at a starting concentration previously determined by the EC_50_ value for around 7–30 weeks depending on the drug, its speed of action and the method used as two methods were considered: high-pressure intermittent selection method and a stepwise selection method. For high pressure selection, cells were treated at a concentration 3–10 × EC_50_ value until more than 95% of the cells died. Then treatment was removed, and cells were allowed to recover. After reaching around 60% semi-confluence, treatment was reinstalled and EC_50_ value monitored. For stepwise selection method, drug concentration used was at the EC_50_ which implied reduced growth rate of approximately 50% and drug pressure was increased in intervals of around 5–10% keeping growth inhibition around 50%. Once the EC_50_ values of the resistant lines were at least 5 times greater than the one used as control, cells were again cloned by limiting dilution and further reconfirmed for drug resistance and subsequent DNA extraction for whole genome sequencing analysis.

### Dose–response assay by EC_50_ determination and bioluminescence quantification

Drug sensitivity and cell viability were assessed by a bioluminescence measurement as follows: 24 h prior to addition of the drugs, 2 × 10^4^ cells/well for every replicate were seeded in a 96-well plate. Once attached, media was removed, and 10 different concentrations of drug were added in serial dilutions 1:3 with a starting concentration of 10 µM or one of which the EC_50_ value of the replicates fell within an intermediate drug concentration. When drug-resistant lines were co-treated in combination with verapamil, a fixed concentration of verapamil (10 µM) was added to every concentration of the drug. After a 48-h incubation period at 37 °C and 5% CO_2_ with the drug, cells were treated with CellTiterGlo (Promega) reagent (diluted 1:2 with deionized water) for quantification of HAP1 cell viability. Immediately after addition of the luminescence reagent, luminescence was measured using the Synergy HT Microplate Reader Siafrtd (BioTek). The data was normalized to 100% cell survival and 100% cell death and EC_50_ values were obtained using the average normalized luminescence intensity of 8 wells per concentration and a non-linear variable slope four-parameter regression curve fitting model in Prism 8 (GraphPad Software Inc.). Unless otherwise noted, dose response experiments consisted of 4–8 technical replicates and 3 biological replicates.

### Isolation of total DNA from drug resistant lines

Genomic DNA (gDNA) was extracted from drug-specific resistant cell lines together with their isogenic parental lines using the DNeasy^®^ Blood & Tissue Kit (Qiagen) following the manufacturer’s instructions. Samples were assessed for quantity with the Qubit™ dsDNA BR Assay Kit (Life Technologies, Carlsbad, CA, USA). All samples (> 2.0 µg, > 50 ng/µL, > 20µL) were prepared for quality control by testing gDNA degradation or potential contamination using agarose gel electrophoresis (1% Agarose, TAE, $\sim$100 Voltage). Then gDNA concentration was again measured using the Qubit^®^ DNA Assay Kit with the Qubit^®^ 2.0 Fluorometer (Life Technologies). Finally, fragment distribution of the gDNA library was measured using the DNA 1000 Assay Kit with the Agilent Bioanalyzer 2100 system (Agilent Technologies, Santa Clara, CA, USA). DNA libraries were sequenced with 150 base pair (bp) paired single end reads on an Illumina HiSeq 4000 (PE150).

### Genome sequencing and data analysis

The quality of the raw FASTQ files was checked with FastQC (http://www.bioinformatics.babraham.ac.uk/projects/fastqc/). Whole genome sequencing (WGS) reads were mapped to GRCh37 (hg19) using BWA (v.0.7.17), specifically with the hs37d5 reference genome from 1000 Genomes project (Phase II). Whole exome sequencing (WES) samples were captured using Agilent SureSelect Human All Exon V6 (58 M), and the reads were also mapped to GRCh37 using BWA (v.0.7.17) with the same reference genome as WGS. Duplicate reads were removed using Picard (v.1.94); paired resistant and parent (WT) BAM files were used as input for The Genome Analysis Toolkit (GATK, v3.8-1). Local realignment and base quality recalibration were performed using default parameters. Somatic single nucleotide variants (SNVs) and small insertion and deletion (indels) were called using GATK MuTect2 following the state-of-the-art GATK Best Practices pipeline (https://ccbr.github.io/Pipeliner/Tools/MuTect2.html). In this project, the input to MuTect2 consisted of alignments for the parent and resistant clone in order to call mutations with statistically significant differences in read support in the setting of resistance. Only the variants with PASS status, suggesting confident somatic mutations, were considered for further analysis. Variant allelic fraction was determined as the fraction of reads supporting the variant allele relative to total read depth at the variant position. Minimum callable depth was set to 10 and base quality score threshold was set to 18, following the default from MuTect2. All sequences have been deposited in SRA BioProject PRJNA603390.

### Small-variant annotation for SNVs and indels

Somatic variants were annotated using snpEff (v 4.3q)^89^. The annotation was performed with parameters including (1) canonical transcripts and (2) protein coding to enable identification of different functional classes of variant and their impact on protein coding genes (Table 1 showing finalized and consolidated annotations; Table S9 shows the raw annotation from snpEff and consolidated classification used in Table 1; Table S5 shows all the SNVs with their raw annotations). The snpEff sequence ontology designation was used in the filtering steps to classify variants generally as noncoding or coding (Table S9).

### Identification of drug specific genes

First, we excluded all variants in non-coding regions. Second, we excluded all non-functional variants, retaining only variants with a snpEff definition of HIGH or MODERATE impact (missense, stop lost, stop gain, and structural interaction variants). Finally, we selected only the variants with high allele frequency (AF > 0.85) and genes with multiple independent amino acid changes found in the same drug as the final list of candidates. The potential candidate variants were evaluated through Integrative Genomics Viewer (IGV)^90^ to ensure coverage and allele fractions of the mutation positions. The top genes for each drug were included in Table 2 and Table S7.

### Somatic copy number variations (CNVs) analysis

Copy number regions for WGS and WES were called by ControlFreeC^47^ using the default settings for WGS and WES data. Background parental clone samples for each drug served as the control. Recurrent CNV regions were defined as regions observed in more than 1 sample, but not in all of clones from the tested drugs (as these are more likely to indicate potential sequencing artifacts).

### Gene knockdowns

shRNAs pools targeting *TOP2A* (Cat# sc-36695-V), *DCK* (Cat# sc-60509-V), *SLCO3A1* (Cat# sc-62713-V), *SLC13A4* (Cat# sc-89760-V), *KLF-1* (Cat# sc-37831-V), *WWOX* (Cat# sc-44193-V), *WDR33* (Cat# sc-94735-V) and the non-coding control (Cat# sc-108080) were obtained in pLKO.1-Puro from Santa Cruz Biotechnology. *RRM1* (clone ID NM_001033.2-476s1c1) and *CYP1B1* (clone ID NM_000104.2-1176s1c1) were obtained in pLKO.1-Puro-CMV-tGFP from Sigma Aldrich.

Gene expression was knocked down using either a shRNA pool (Santa Cruz Biotechnology) containing between three and five expression constructs each encoding target-specific 19–25 shRNAs or a single shRNA (Sigma Aldrich). HAP1 cells were plated at 120,000 cells per well (~ 40% confluency) in a 24-well plate, 24 h prior to viral transduction. On the day of transduction, complete media was replaced with serum-free media and 7 µg/mL Polybrene^®^ (Cat# sc-134220) and virus was added to the cells at a multiplicity of infection of 0.5 and cells were incubated overnight at 37 °C. The following day, media was replaced with complete media without Polybrene and cells were incubated at 37 °C overnight. Cells were then split 1:3 and incubated for 24 h more and finally stable clones expressing the shRNA were selected using complete media with 2 µg/mL puromycin. After 7 days of selection with puromycin, knockdown efficiency was confirmed by western blot. Cells transduced with shRNAs containing fluorescent tags, were trypsinized (TrypLE™ Express; Cat# 12605-010, Gibco) after puromycin selection, washed twice with DPBS (1X) (Gibco) and sorted by flow cytometry.

### Knockout of USP47

*USP47* was knocked out (Cat# HSPD0000092816) using a single plasmid CRISPR-*Cas9* system, using as lentivirus backbone the LV01 U6-gRNA:ef1a-puro-2A-Cas9-2A-tGFP targeting *USP47* (Sigma Aldrich). The target sequence (5ʹ–3ʹ) was CAATGGGGCTTCTACTAGG. Transduction was as described above. HAP1 cells were plated at 120,000 cells per well (~ 40% confluency) in a 24-well plate 24 h prior to viral transduction. On the day of transduction, complete media was replaced with serum-free media and 7 µg/mL Polybrene^®^ (Cat# sc-134220), virus was added to the cells at a multiplicity of infection of 0.5 and cells were incubated overnight at 37 °C. The following day, media was replaced with complete media without Polybrene and cells were incubated at 37 °C overnight. Cells were then split 1:3 for 24 h more and stable clones expressing the CRISPR-*Cas9* sequence were selected using complete media with 2 µg/mL puromycin. After 14 days of selection with puromycin and propagation as required, cells were trypsinized (TrypLE™ Express; Cat# 12605-010, Gibco), washed twice with DPBS (1X) (Gibco) and sorted by flow cytometry using the GFP fluorochrome which is expressed with Cas9. GFP positive cells were plated at an average density of 0.5 cells per well in a 96-well plate (previously treated with poly-l-Lysine (Sigma #P4707-50 ml) to improve cell adhesion) in presence of 2 µg/mL puromycin (limiting dilution cloning). Cell growth was monitored via microscopy during 25 days to select those wells which were observed to contain single colonies and *USP47* knockout was confirmed in those monoclonal HAP1 cell lines first via PCR and then reconfirmed by western blot using the *USP47* rabbit polyclonal antibody (Abcam, Cat# ab97835).

### Immunoblotting

HAP1 cells (at least 5 × 10^6^) were trypsinized, washed twice with cold 1 × DPBS and then lysed in 500 µL Pierce™ RIPA Buffer (Thermo Scientific) containing 1:100 protease inhibitor (Halt™ Protease & Phosphatase Inhibitor Cocktail, Thermo Scientific) and 1:100 0.5 M EDTA Solution (Thermo Scientific). Total protein concentration was measured using the DC Protein Assay (Bio-Rad). Equal amounts of proteins were resolved by SDS-PAGE and transferred to nitrocellulose membranes (Bio-Rad #1704271), blocked in PBS with 5% (w/v) Blotting-Grade Blocker (Bio-Rad #170-6404) and 0.1% (v/v) Tween20 for 1 h and probed. As secondary antibodies, HRP-linked anti-mouse or anti-rabbit (Cell Signaling Technology) were used and the HRP signal was visualized with SuperSignal®West Pico Chemiluminescent Substrate (Thermo Scientific #34080) using Syngene G-Box imager. Protein enrichment was calculated relative to *vinculin*, *γ-tubulin* or *β-actin*. Primary antibodies are listed below. Full size western blots are shown in Fig. S9.

### Antibodies

*TOP2A* (Sigma #SAB4502997), *USP47* (Abcam #ab97835), *WDR33* (Abcam #ab72115), *DCK* (Abcam #ab151966), *β-actin* (Cell Signaling #3700S), *γ-tubulin* (Cell Signaling #4285S), *Vinculin* (Invitrogen #700062), *SLC13A4/SUT-1* (Abcam #ab236619), *WWOX* (Abcam #ab137726), *EKLF/KLF-1* (Abcam #175372), *SLCO3A1/OATP-A* (Santa Cruz #sc-365007), *TOP1* (Proteintech #20705-1-AP), CRISPR-*Cas9* (Sigma #SAB4200701), *RRM1* (Abcam #ab133690), *CYP1B1* (Abcam #ab137562), *SPG7* (Sigma #SAB1406470 and Abcam #ab96213), goat anti-mouse (Invitrogen #G21040), goat anti-rabbit (Invitrogen #G21234).

### RNA isolation, RT-PCR analysis and sequencing of TOP1 (*His81*)

TPT-resistant cells and TPT-WT (1 × 10^6^ cells) were dissociated from plates by the addition of 2 mL of TrypLE (Cat #12605-010, Gibco), washed and total RNA was isolated and purified using a Qiagen RNeasy® Mini Kit (Cat #74104, Qiagen) according to manufacturer’s instructions. cDNA was synthesized from 1 µg of total RNA using the Superscript™ First-Strand Synthesis System for RT-PCR Kit (Invitrogen #11904-018) and random hexamers. The primers used to amplify the region containing *His81* were FWD: GATCGAGAACACCGGCAC and REV: TCAGCATCATCCTCATCTCGAG. DNA from PCR product was extracted, using the QIAquick® Gel Extraction Kit (Qiagen #28706) following the manufacturer’s instruction, measured using the Qubit® DNA Assay Kit with the Qubit® 2.0 Fluorometer (Life Technologies), and sequenced. The cDNA was sent to Eton Biosciences for Sanger sequencing. Quantification of TOP1 expression was performed using PerfeCTa® Sybr Green Fast Mix (Quanta #95072-250) the following primers: FWD: CGAATCATGCCCGAGGATATAA; REV: CCAGGAAACCAGCCAAGTAA, following the manufacturer’s instruction.

### Supplementary Information

Supplementary Information.

Supplementary Table S3.

Supplementary Table S4.

Supplementary Table S5.

Supplementary Table S7.

Supplementary Table S8.

## Data Availability

All whole genome sequences have been deposited in SRA BioProject PRJNA603390.

## Abbreviations

AF: Allele frequency
CNV: Copy number variation
NGS: Next generation sequencing
WES: Whole exome sequencing
WGS: Whole genome sequencing
CML: Chronic myelogenous leukemia
IVIEWGA: In vitro evolution and whole genome analysis
SNV: Single nucleotide variant
TF: Transcription factor
DOX: Doxorubicin
GEM: Gemcitabine
ETP: Etoposide
PTX: Paclitaxel
TPT: Topotecan
AML: Acute myeloid leukemia
TKIs: Tyrosine kinase inhibitors
MDR: Multi-drug resistance
gDNA: Genomic DNA
