# Supplementary material for: In vitro evolution and whole genome analysis to study chemotherapy drug resistance in haploid human cells
Source: Sci Rep. 2024 Jun 18;14:13989. doi: 10.1038/s41598-024-63943-7 (PMC11183241; doi:10.1038/s41598-024-63943-7)
Supplement: Supplementary file 1 — Supplementary Information. [file 41598_2024_63943_MOESM1_ESM.docx]

Supplemental files

In vitro evolution and whole genome analysis to study drug resistance in haploid human cells

Juan Carlos Jado^1,2,*^, Michelle Dow^2,3,4,*^, Krypton Carolino^5^, Adam Klie^2^, Gregory J. Fonseca^6^, ^.^Trey Ideker^2,7,#^, Hannah Carter^2,7,#^, Elizabeth A. Winzeler^1,2,#^

**Affiliations:**

^1^Division of Host-Microbe Systems & Therapeutics, Department of Pediatrics, University of California, San Diego, Gilman Dr., La Jolla, CA 92093, USA

^2^Department of Medicine, Division of Medical Genetics, University of California San Diego, La Jolla, CA 92093, USA

^3^Bioinformatics and Systems Biology Graduate Program, University of California San Diego, La Jolla, CA 92093, USA

^4^Health Science, Department of Biomedical Informatics, School of Medicine, University of California San Diego, La Jolla, CA 92093, USA

^5^Division of Biological Sciences, University of California San Diego, La Jolla, CA 92093, USA

^6^Department of Medicine, Meakins-Christie Laboratories, McGill University Health Centre, 1001 Decaire Blvd, Montreal, QC H4A 3J1, McGill University, Montreal, Quebec, Canada

^7^Moores Cancer Center, University of California San Diego, La Jolla, CA 92093, USA

*These authors contributed equally

^#^Correspondence to Elizabeth Winzeler (ewinzeler@ucsd.edu) and Hannah Carter (hcarter@ucsd.edu)

Table of Contents

| [Table S1. Summary of all drugs tested for IVIEWGA. 3](#_Toc165010659)  [Table S2. EC_50_ values for all drug-specific resistant lines and their isogenic parental cell line (WT). 5](#_Toc165010660)  [Table S3. Sequencing sample characteristics and statistics (attached Excel file). 5](#_Toc165010661)  [Table S4. Summary of mutation types for each individual clone (attached Excel file). 5](#_Toc165010662)  [Table S5. All SNVs called from the 28 samples (attached Excel file). 5](#_Toc165010663)  [Table S6. Potential target genes fitting the filtering criteria. 6](#_Toc165010664)  [Table S7. Genes that contain independent disruptive mutation or amplification in one or more sequenced lines (attached Excel file). 7](#_Toc165010665)  [Table S8. All CNVs called from the 28 samples (attached Excel file). 7](#_Toc165010666)  [Table S9. A. Summary of effect classification of the SNVs and Indels from snpEff annotations. 8](#_Toc165010667)  [Figure S1. Hierarchical tree of the parent clones and the different drug-specific replicates. 9](#_Toc165010668)  [Figure S2. Near-normal distribution of the mutations in respect to chromosome length. 10](#_Toc165010669)  [Figure S3. Mutation filtering pipeline strategies and quantitative summary. 11](#_Toc165010670)  [Figure S4. A. CNV events across samples in each chromosome for WGS and WES samples. 12](#_Toc165010671)  [Figure S5. Cross-drug resistance EC_50_ curves. 13](#_Toc165010672)  [Figure S6. ETP target genes and validation results. 14](#_Toc165010673)  [Figure S7. IGV views for the TPT target genes. 15](#_Toc165010674)  [Figure S8. RT-qPCR quantifying expression of TOP1 in TPT-WT, TPT-R4a-c resistant lines. 16](#_Toc165010675)  [Figure S9. Western Blots (full membrane) for validated genes. 17](#_Toc165010676) |
| --- |


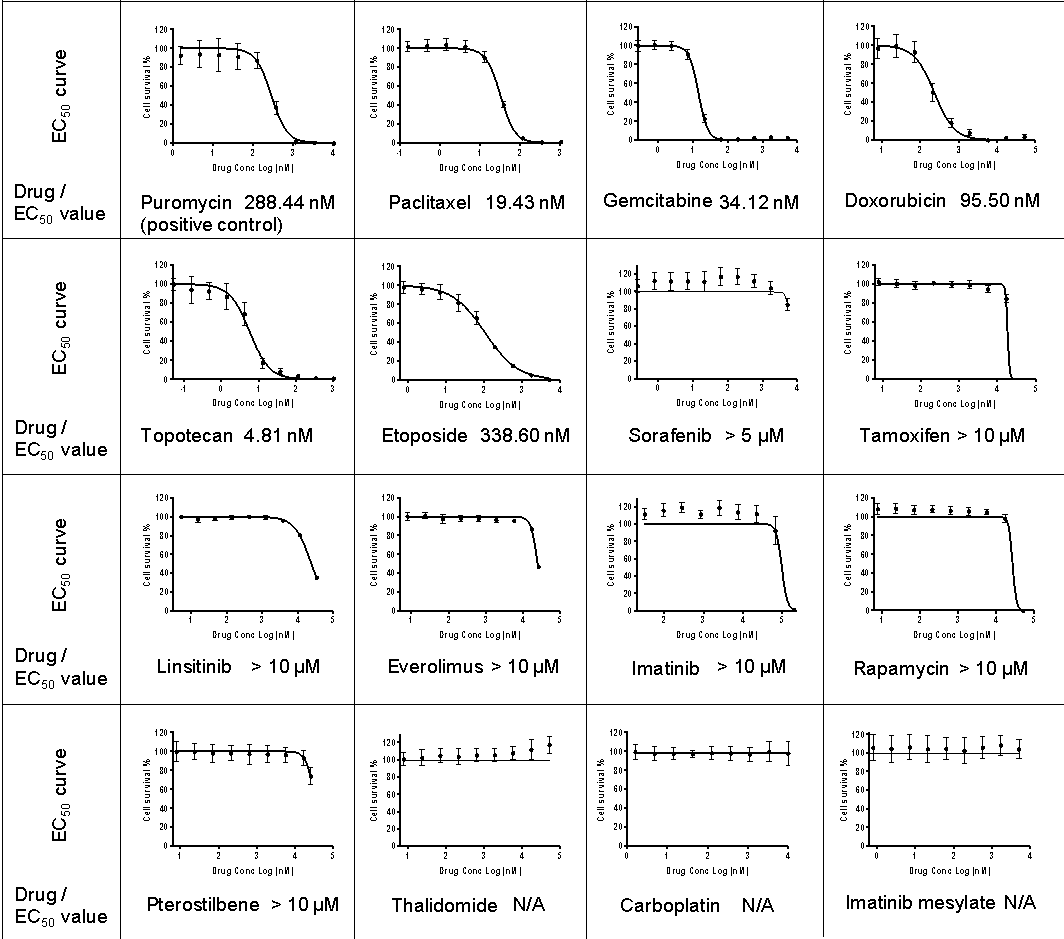


# Table S1. Summary of all drugs tested for IVIEWGA.

Different anticancer FDA-approved drugs were tested to determine their EC_50_. ATP levels were measured via bioluminescence (CellTiterGlo) to determine drug sensitivity in a dose-response assay for 48h with serial dilutions of the drug (10µM max, 1:3 serial dilutions, 8 technical replicates per concentration point). Those drugs showing EC_50_ values below 1µM were considered for IVIEWGA. Puromycin was used as a positive control.

|  |  | BR1 | BR2 | BR3 | AVRG | STDEV |
| --- | --- | --- | --- | --- | --- | --- |
| Doxorubicin | DOX-WT1 | 45.2 | 51.0 | 41.9 | 46.1 | 4.6 |
|  | DOX-R1 | 840.9 | 1144 | 945 | 976.6 | 154 |
|  | DOX-R2 | 833.7 | 1106 | 957.8 | 965.8 | 136.3 |
|  | DOX-R3 | 1132 | 1292 | 1225 | 1216.3 | 80.4 |
|  | DOX-WT5 | 150 | 150.6 | 134.2 | 144.9 | 9.3 |
|  | DOX-R4a | 1373 | 1403 | 1162 | 1312.7 | 131.3 |
|  | DOX-R4b | 503.4 | 504.5 | 466.6 | 491.5 | 21.6 |
|  | DOX-R5 | 1510 | 1513 | 1455 | 1492.7 | 32.7 |
| GEMCITABINE | GEM-WT2 | 59.3 | 61.5 | 57.8 | 59.5 | 1.9 |
|  | GEM-R1 | 21774 | 22069 | 20377 | 21406.7 | 903.8 |
|  | GEM-R2 | 19756 | 19917 | 16721 | 18798 | 1800.5 |
|  | GEM-R3 | 27722 | 25236 | 20529 | 24495.7 | 3653.2 |
|  | GEM-WT3 | 9.4 | 8.8 | 8.1 | 8.7 | 0.7 |
|  | GEM-R4 | 60.1 | 58.9 | 45.3 | 54.8 | 8.2 |
|  | GEM-R5 | 82.1 | 78.3 | 66.1 | 75.5 | 8.4 |
|  | GEM-R6 | 33.6 | 28.1 | 24.3 | 28.7 | 4.6 |
| PACLITAXEL | PTX-WT4 | 22.8 | 20.6 | 21.2 | 21.5 | 1.2 |
|  | PTX-R1 | 120.6 | 231.5 | 251.4 | 201.2 | 70.5 |
|  | PTX-R2a | 89.4 | 97.9 | 102.9 | 96.7 | 6.8 |
|  | PTX-R2b | 152.9 | 255 | 215.3 | 207.7 | 51.5 |
|  | PTX-R3 | 208.9 | 283.9 | 221 | 237.9 | 40.3 |
|  | PTX-WT5 | 20.4 | 19.3 | 13 | 17.5 | 4 |
|  | PTX-R4 | 855.3 | 959.1 | 843.9 | 886.1 | 63.5 |
|  | PTX-R5 | 824.8 | 763.6 | 659.3 | 749.2 | 83.7 |
|  | PTX-R6 | 1018 | 1073 | 981.7 | 1024.2 | 46 |
| Topotecan | TPT-WT6 | 5.6 | 5.7 | 5.6 | 5.6 | 0.1 |
|  | TPT-R1 | 140.8 | 150.4 | 79.9 | 123.7 | 38.2 |
|  | TPT-R2 | 165.7 | 299 | 169.7 | 211.5 | 75.8 |
|  | TPT-R3 | 211.2 | 291.2 | 177.2 | 226.5 | 58.5 |
|  | TPT-WT7 | 3.4 | 3.7 | 3.6 | 3.6 | 0.2 |
|  | TPT-R4a | 34.8 | 23.9 | 29.4 | 29.4 | 7.7 |
|  | TPT-R4b | 59.2 | 42.1 | 51.5 | 50.9 | 12.1 |
|  | TPT-R4c | 68.9 | 64.9 | 66.9 | 66.9 | 2.8 |
| ETOP. | t-WT3 | 292.8 | 363.6 | 359.4 | 338.6 | 39.7 |
|  | ETP-R1 | 5876 | 9851 | 8235 | 7987.3 | 1999 |
|  | ETP-R2 | 4230 | 5496 | 4010 | 4578.7 | 802 |
|  | ETP-R3 | 3742 | 3022 | 2715 | 3159.7 | 527.2 |

# Table S2. EC_50_ values for all drug-specific resistant lines and their isogenic parental cell line (WT).

EC_50_ averaged values (AVRG) are presented as mean of 4-8 technical replicates with individual biological replicates (BR) overlaid. Doxorubicin (DOX), Gemcitabine (GEM), Paclitaxel (PTX), Topotecan (TPT) and Etoposide (ETP) were used to generate resistant lines as described in methods.

# Table S3. Sequencing sample characteristics and statistics (attached Excel file).

# Table S4. Summary of mutation types for each individual clone (attached Excel file).

# Table S5. All SNVs called from the 28 samples (attached Excel file).

| Gene | Sample | Type | Amino acid change | AF |
| --- | --- | --- | --- | --- |
| *AC091801.1* | DOX-R4a | MS | His13Asn | 1 |
| *TOP2A* | DOX-R2, DOX-R3 | MS | Pro803Thr | 0.89, 0.87 |
| *EDNRA* | DOX-R2, DOX-R3 | MS | Ala143Ser | 0.86, 0.88 |
| *ITGB3* | DOX-R2, DOX-R3 | MS | Cys627Phe | 0.88, 0.96 |
| *PRAMEF11* | DOX-R2, DOX-R3 | MS | Gln377Lys | 0.91, 0.90 |
| *NCAN* | DOX-R3 | MS | G939Ser | 0.97 |
| *TRIM45* | DOX-R3 | MS | Gln305Lys | 0.89 |
| *SLC13A4* | DOX-R4b | MS | Gly165His | 1 |
| *KCNC3* | DOX-R5 | MS | Ser379Ile | 0.86 |
| *SPG7* | DOX-R5 | MS | Lys593Asn | 1 |
| *WDR87* | DOX-R5 | MS | Arg2418Met | 0.89 |
| *WDR33* | ETP-R3 | MS | Pro622Thr | 1 |
| *STARD9* | GEM-R4, GEM-R5, GEM-R6 | MS | His1021Tyr (R4, R5, R6), Ser1330Ile (R5) | 0.48, 0.47, 0.45, 0.33 |
| *SLCO3A1* | PTX-R2b, PTX-R6 | MS | Ile587Asn (R2b), Ala263Thr (R6) | 0.23, 0.07 |
| *CYP1B1* | TPT-R1, TPT-R4a, TPT-R4b, TPT-R4c | MS | Val432Leu, Asp217Glu (R4a, R4b, R4c) | 0.13, 0.40, 0.43, 0.42 |
| *TOP1* | TPT-R4a, TPT-R4b, TPT-R4c | FS | His81fs | 1 |
| *CEACAM1* | TPT-R4c | MS | Asp479Glu | 1 |
| *VAMP7* | TPT-R4c | MS | Gln57Lys | 1 |
| *ZFP36* | TPT-R4c | MS | Asp260Tyr | 1 |
| *DCK* | GEM-R1, GEM-R2, GEM-R3 | MS, FS | Ser129Tyr (R1, R2), Asn80fs (R1. R3), Asn113fs (R2), Thr184fs (R3) | 0.78, 0.25; 0.28, 0.43; 0.09, 0.14 |
| *GRM3* | ETP-R3 | SIV | - | 1 |

# Table S6. Potential target genes fitting the filtering criteria.

Listed genes are the completed set of mutations that presented with AF > 0.85, and modified the coding potential, or were mutated multiple times at independent samples. AF, allele frequency; CNV, copy number variant; MS, missense; SIV, structural variant; FS, frameshift.

# Table S7. Genes that contain independent disruptive mutation or amplification in one or more sequenced lines (attached Excel file).

# Table S8. All CNVs called from the 28 samples (attached Excel file).

| Noncoding Variants | |
| --- | --- |
| *SnpEff Annotation* | *Effect Classification* |
| 3_prime_UTR_variant | Intergenic |
| 5_prime_UTR_premature_start_codon_gain_variant | Intergenic |
| 5_prime_UTR_variant | Intergenic |
| TF binding site variant | Intergenic |
| Conservative inframe deletion | Inframe deletion |
| Conservative inframe insertion | Inframe insertion |
| Disruptive inframe deletion | Disruptive inframe deletion |
| Disruptive inframe insertion | Disruptive inframe insertion |
| Downstream gene variant | Intergenic |
| Intergenic region | Intergenic |
| Intragenic variant | Intragenic |
| Intron variant | Intron |
| Sequence feature | Intergenic |
| Splice acceptor variant & intron variant | Splice region plus intron variant |
| Splice acceptor variant & splice donor variant & splice region variant & 5 prime UTR variant & intron variant | Splice region plus intron variant |
| Splice acceptor variant & splice donor variant & splice region variant & intron variant | Splice region plus intron variant |
| Splice acceptor variant & splice region variant & conservative inframe deletion & intron variant | Splice region plus intron variant |
| Splice acceptor variant & splice region variant & disruptive inframe deletion & intron variant | Splice region plus intron variant |
| Splice donor variant & intron variant | Splice region plus intron variant |
| Splice region variant | Splice region plus intron variant |
| Splice region variant & intron variant | Splice region plus intron variant |
| Splice region variant & synonymous variant | Splice region plus intron variant |
| Coding Variants | |
| *SnpEff Annotation* | *Effect Classification* |
| Frameshift variant | Frameshift |
| Frameshift variant & splice acceptor variant & splice donor variant & splice region variant & intron variant | Frameshift |
| Frameshift variant & splice acceptor variant & splice region variant & intron variant | Frameshift |
| Frameshift variant & splice region variant | Frameshift |
| Frameshift variant & stop gained | Frameshift plus stop-gained |
| Missense variant | Missense |
| Missense variant & splice region variant | Missense |
| Protein-protein contact | Other nonsynonymous coding |
| Start lost | Start lost |
| Stop gained | Stop gained |
| Stop lost | Stop lost |
| Structural interaction variant | Other nonsynonymous coding |
| Synonymous variant | Synonymous |

# Table S9. A. Summary of effect classification of the SNVs and Indels from snpEff annotations.


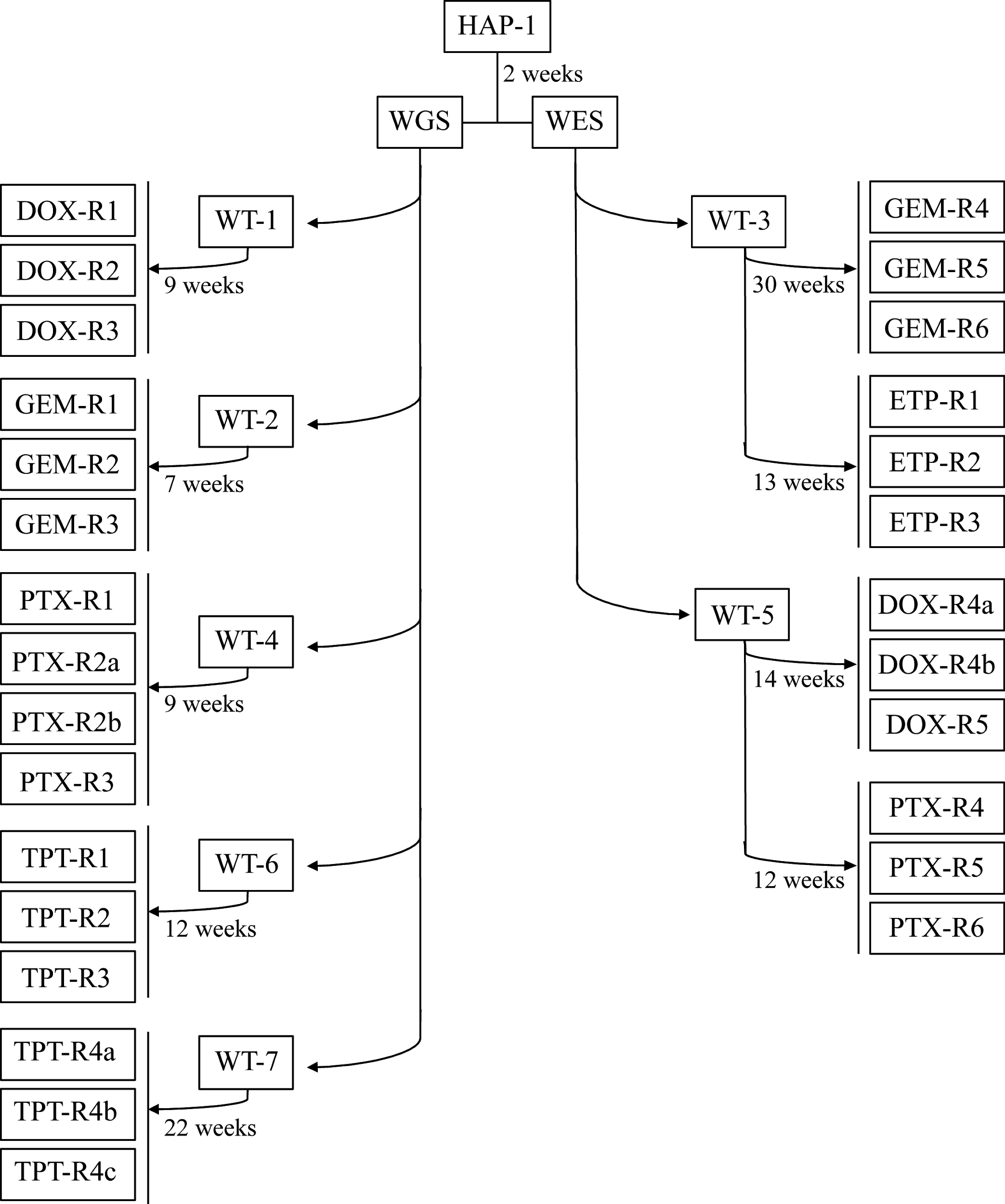


# Figure S1. Hierarchical tree of the parent clones and the different drug-specific replicates.

At least 3 independent HAP-1 drug-resistant clones were generated directly from their isogenic wildtype parents (WT) and their DNA was whole genome (WGS) or whole exome (WES) sequenced. The independent HAP-1 clones were subjected to increasing sublethal concentrations of the drugs based on the starting EC_50_ values for each chemotherapeutic agent until they acquired resistance. The anticancer drugs used for the study were doxorubicin (DOX), gemcitabine (GEM), paclitaxel (PTX), topotecan (TPT) and etoposide (ETP). The time required to generate drug-resistant clones varied depending on the drug from 7 weeks up to 30 weeks (49 to 210 generations). In a few cases, independent selections could not be achieved and dependent clones with a shared lineage were collected.


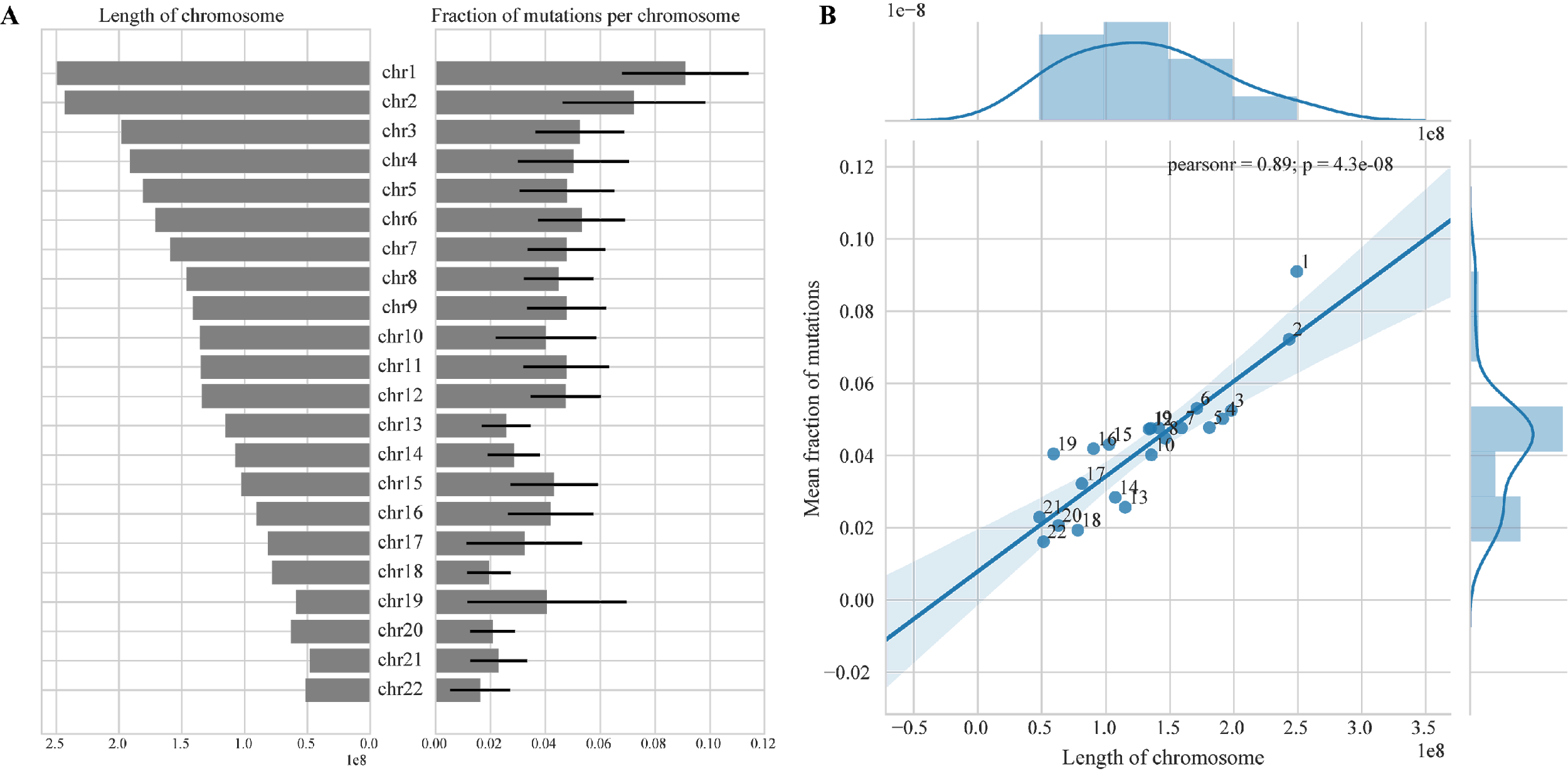


# Figure S2. Near-normal distribution of the mutations in respect to chromosome length.

A. A bar plot showing length of chromosomes (left) and the fraction of mutations (right) across all samples for each chromosome. Error bars show the standard deviation between samples for their fraction of mutations. B. The length of chromosome (x-axis) shows a 0.89 correlation to the mean fraction of mutations (y-axis) for the samples.

**
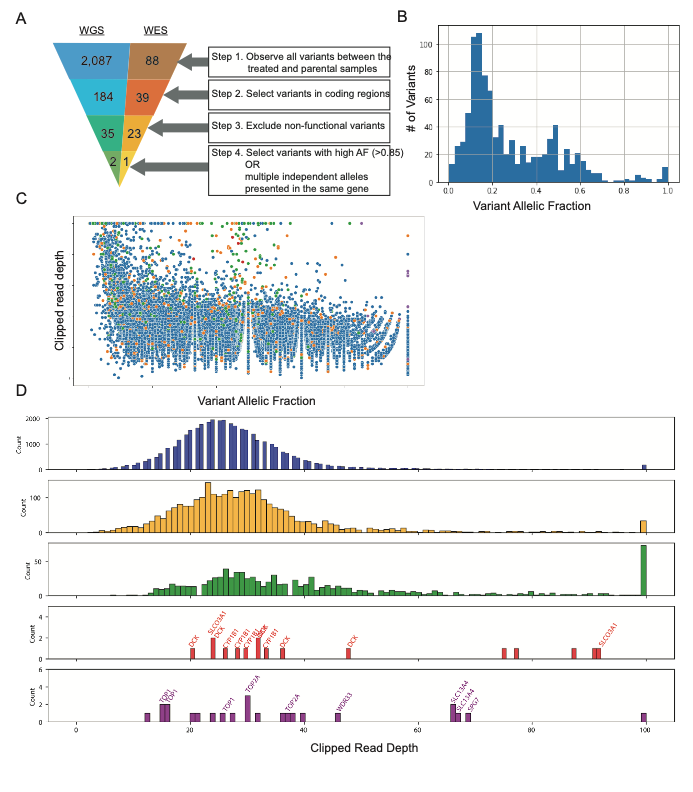
**

Figure S3. Mutation filtering pipeline strategies and quantitative summary.

**A.** Variant filtering pipeline and the average number of mutations per WGS and WES samples. Allelic fraction versus read depth for candidate resistance variants. **B.** Histogram of allelic fractions of coding and functional variants detected in HAP-1 IVIEWGA study. **C.** Scatter plot showing the relationship between read depth and allelic fraction across all variants detected. **D.** Histograms showing the read depth of variants in each category. Variant sites for final selected targets generally had a read depth of 20 or higher.

**
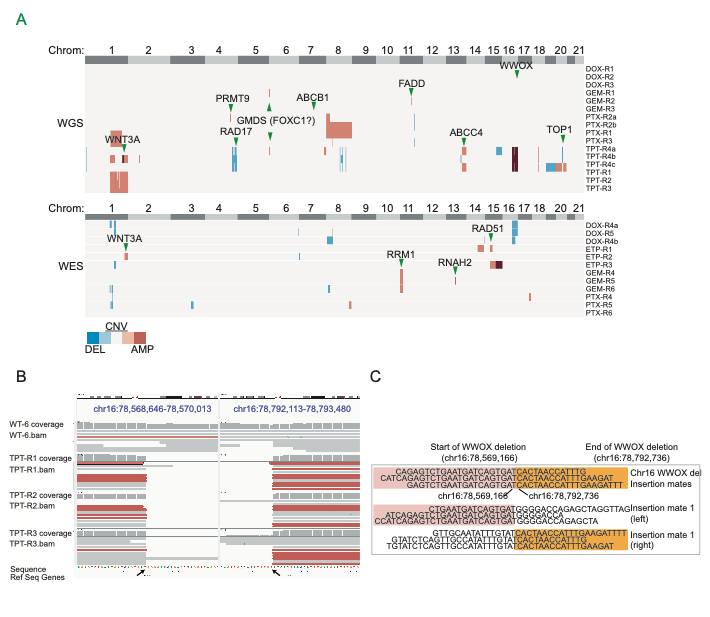
**

Figure S4. A. CNV events across samples in each chromosome for WGS and WES samples. Events less than 0.05% (5E-04) of the chromosome length and events from chromosome X and Y are not shown. The full list of CNV events can be found in Table S7. Candidate genes in small CNVs were identified by examining protein coding genes in minimal amplified intervals to look for classes of genes known to be associated with resistance (ABC transporters, DNA damage response and cell cycle, cytochrome p450s) and performing literature searches on potential candidates. **B.** IGV screen view of the *WWOX* deletion event showing the start (left) and end (right) of the deletion as well as a histogram of coverage at each base. Reads with an abnormal paired-end insert size (~223,000 versus ~200 bases) are highlighted in red. **C.** Portions of reads covering the start and end of the deletion events are shown and allow the identification of the exact breakpoint.


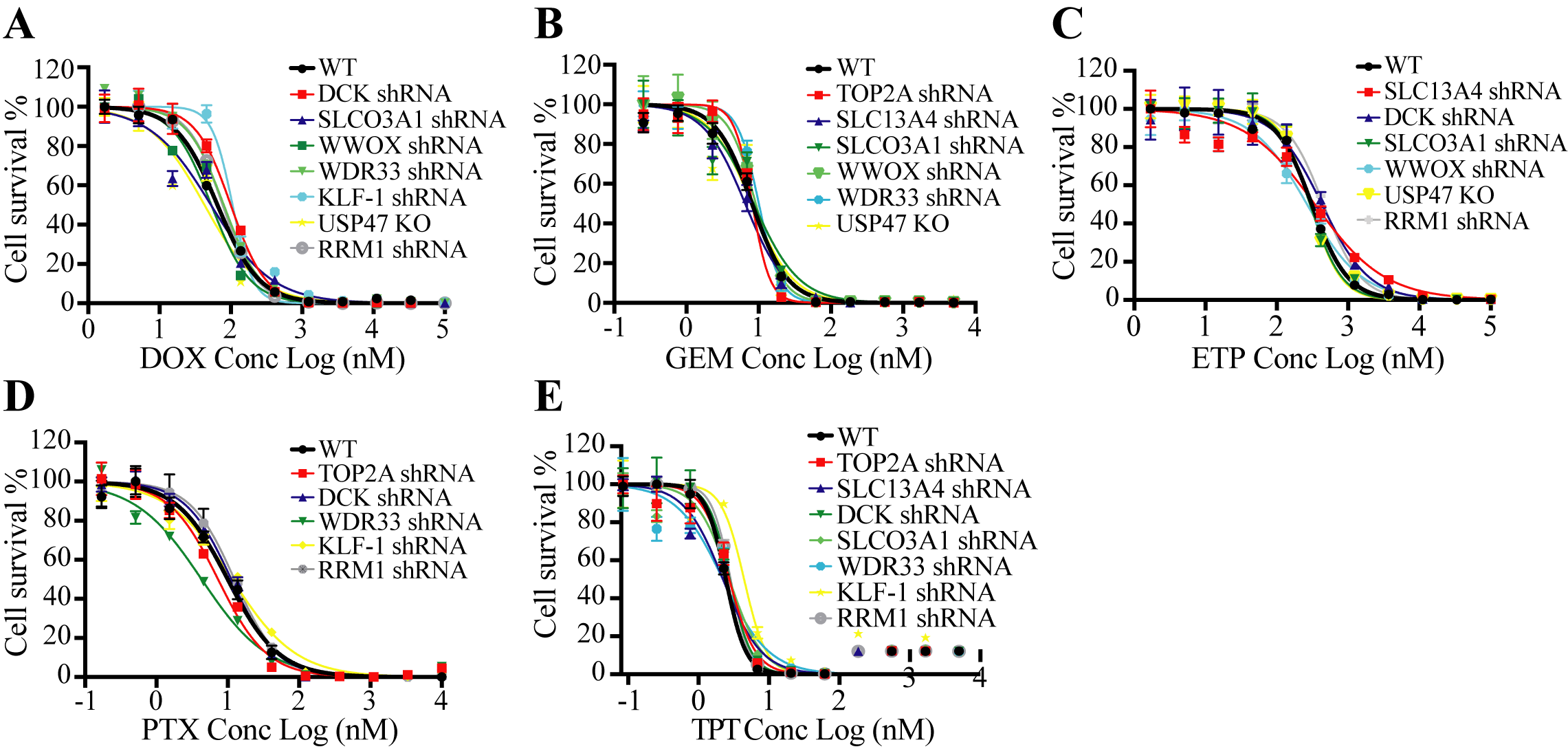


# Figure S5. Cross-drug resistance EC_50_ curves.

EC_50_ curves for all validated gene candidates that showed absence of multidrug resistance (MDR) pathways against the other drugs used in the study. Target gene candidates tested against: A. DOX, B. GEM, C. ETP, D. PTX, and E. TPT. Experiments were conducted in technical quadruplicates.


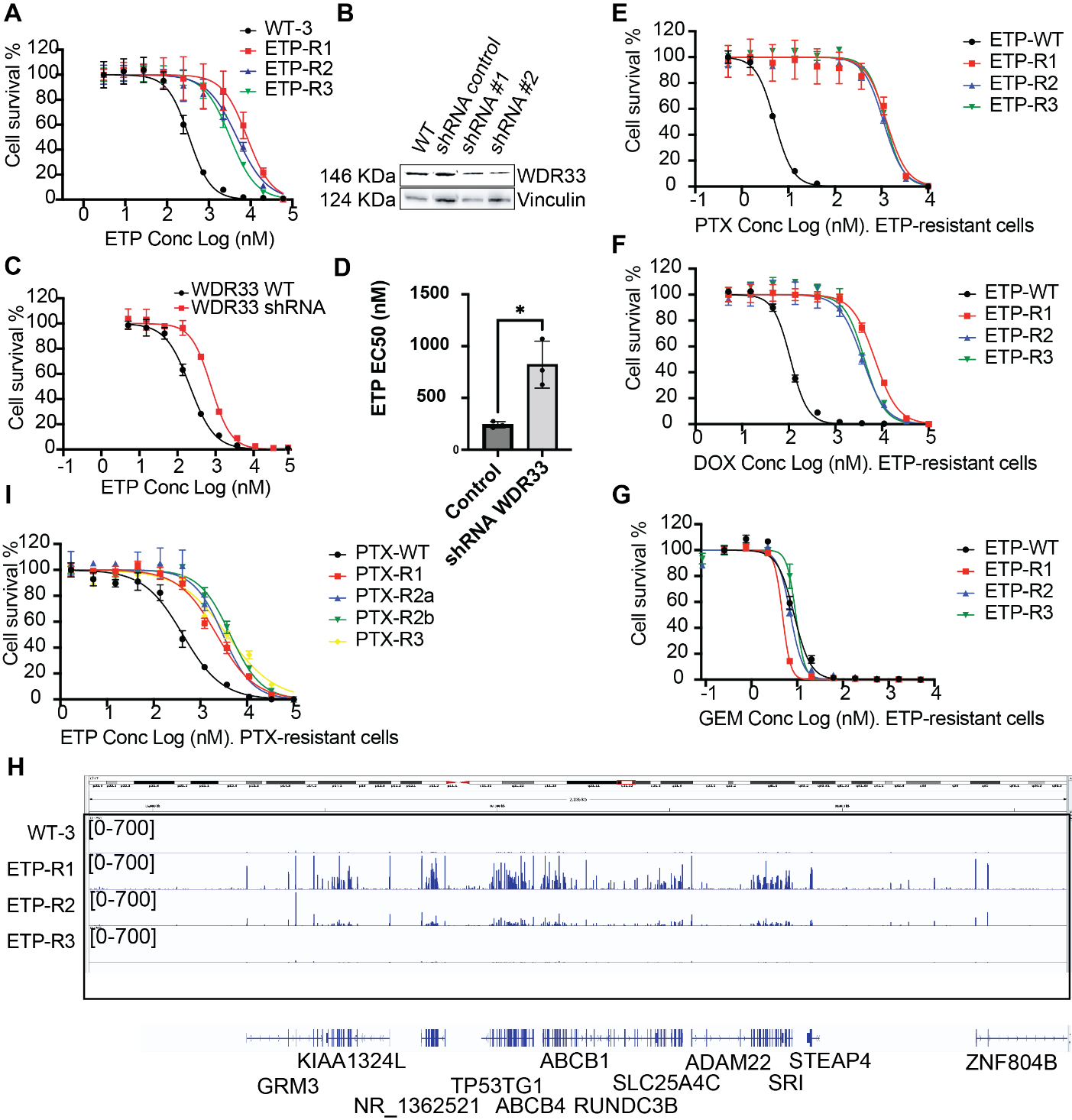


# Figure S6. ETP target genes and validation results.

A. EC_50_ curves for initial screening of ETP resistance. B. Western blot with their gene depletion downregulates protein levels for *WDR33*. C. EC_50_ curves of the WT and shRNA knock-down cell lines. D. Boxplot of the WT and shRNA knockdown cell lines for *WDR33*. E. EC_50_ curves for cross resistance screening of ETP lines against PTX. F. EC_50_ curves for cross resistance screening of ETP lines against DOX G. EC_50_ curves for cross resistance screening of ETP lines against GEM. H. IGV view of ABCB1 region for parental and ETP resistant lines. Read count ranges are shown on left in brackets. I. EC_50_ curves cross resistance screening of PTX lines against ETP. Data is represented by mean ± s.e.m. with n=3 biological replicates overlaid and n=4-8 technical replicates. ** = p value < 0.01. p values determined by two-tailed *t* test.


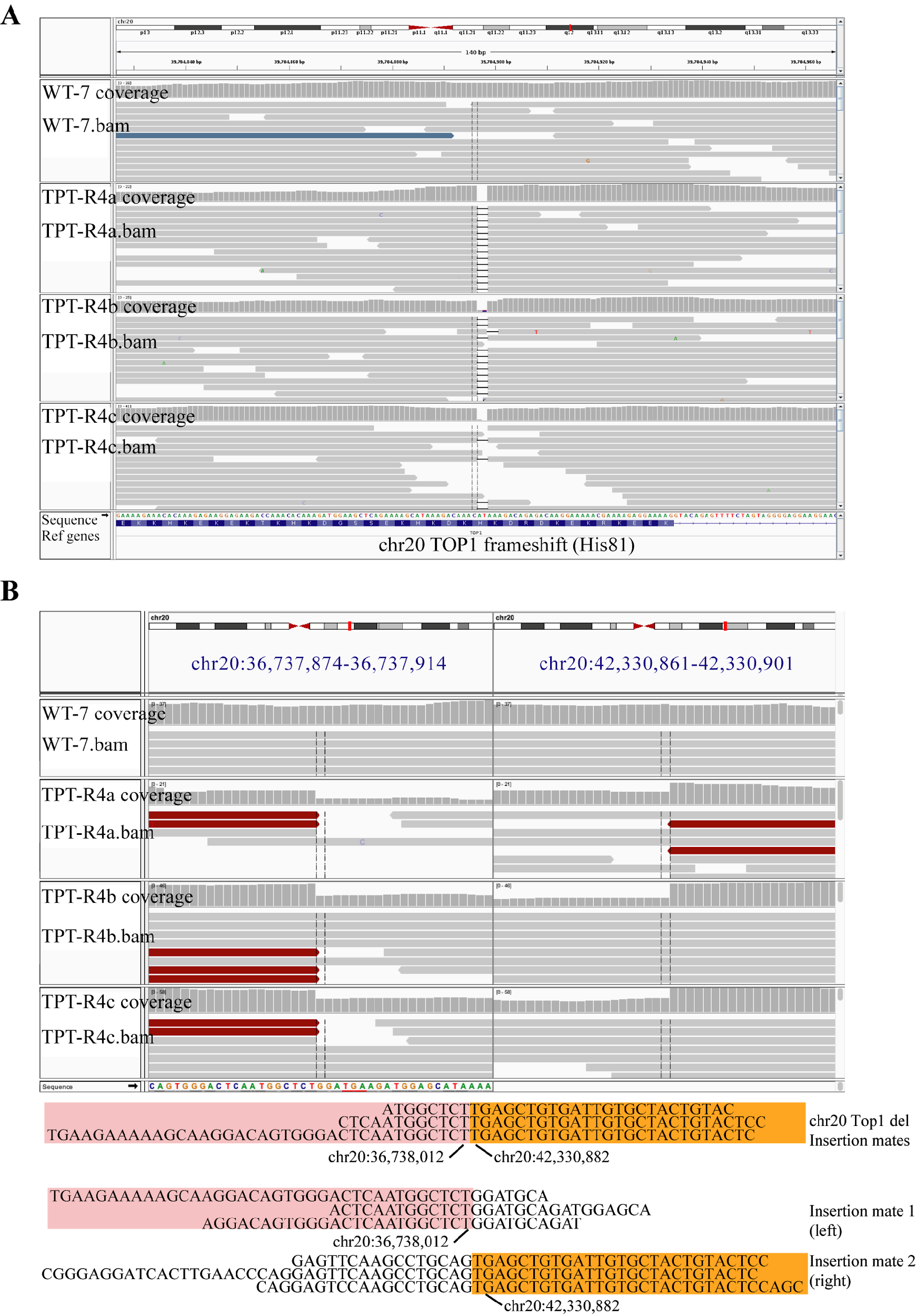


# Figure S7. IGV views for the TPT target genes.

A. IGV view of *TOP1*frameshift (His81) at TPT cell lines. B. IGV view of the start of the deletion event that includes *TOP1,*where the reads dropped at TPT-R4a, TPT-R4b, and TPT-R4c cell lines compared to WT-7. Left shows the start of the deletion event and right shows the end. At the drop of the reads, there are also insertion events in the replicates but not in the WT.

**
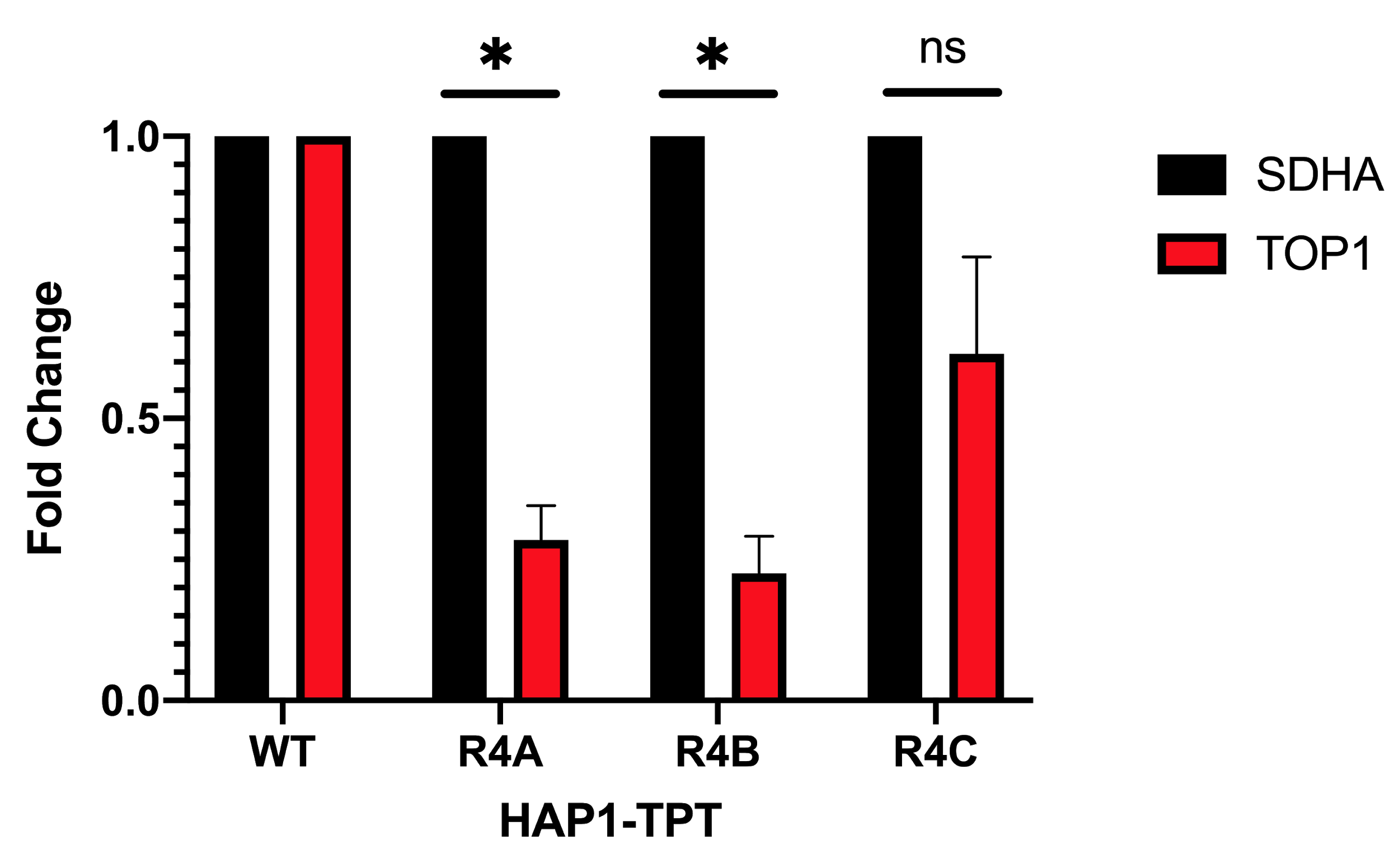
**

# Figure S8. RT-qPCR quantifying expression of TOP1 in TPT-WT, TPT-R4a-c resistant lines.

HAP1 wild type and TPT-R4a-c resistant lines were subject to RNA extraction, followed by cDNA generation using random hexamers. Then, qPCR was utilized to measure mRNA expression level of TOP1 in each line relative to the succinate dehydrogenase (SDHA) controls.


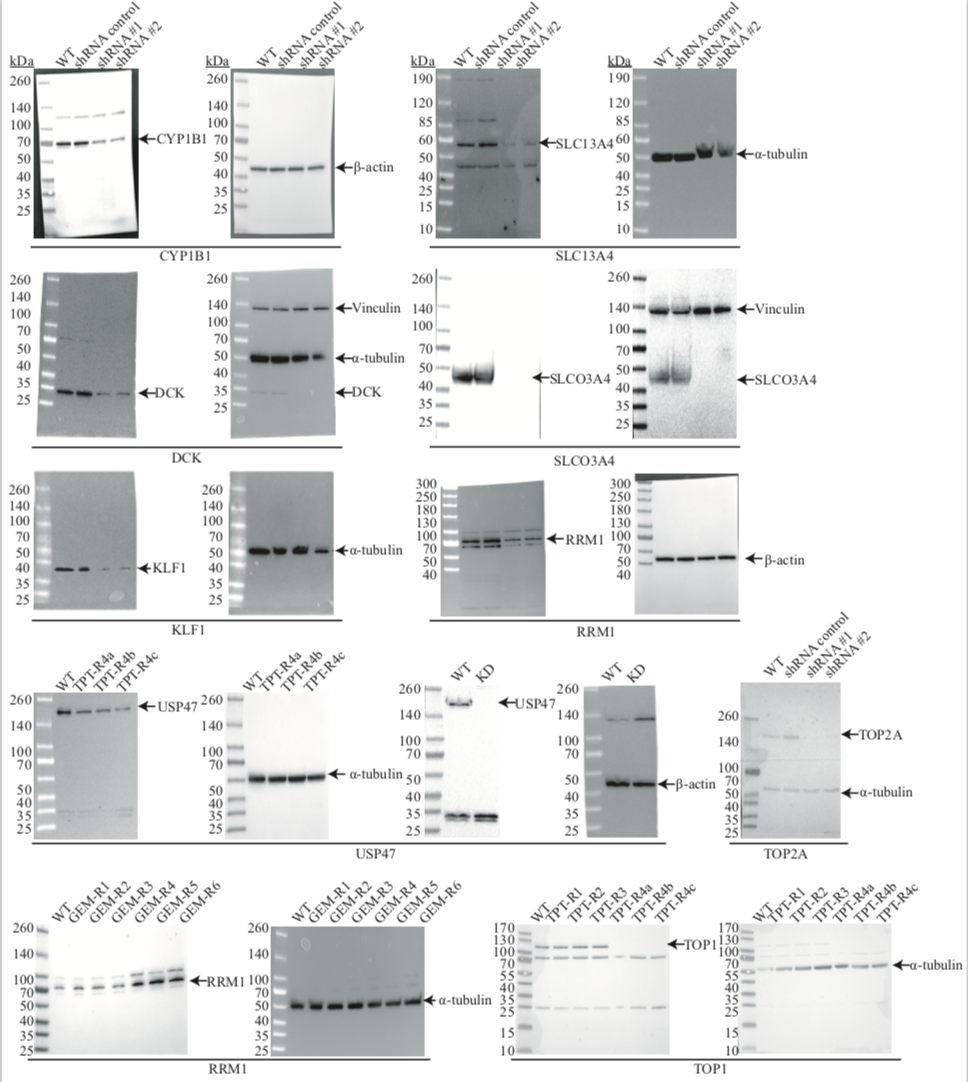


# Figure S9. Western Blots (full membrane) for validated genes.

Gene depletion via shRNA or CRISPR/Cas9 of all 10 validated genes as well as overexpression or downregulation of the drug-resistant clones compared to their isogenic parents and the scrambled controls are shown in whole membrane together with their corresponding loading controls. Deoxycytidine kinase (*DCK*), Krüppel-Like Factor 1 (*KLF-1*), Topoisomerase II alpha (*TOP2A*), solute carrier transporters (*SLC13A4* and *SLCO3A1*), the ubiquitin-specific peptidase *USP4*7, *WDR33*, tumor suppressor *WWOX*, the catalytic subunit of ribonucleotide reductase *RRM1* and the cytochrome p450 *CYP1B1* were considered in our study. *Vinculin*, *γ-tubulin* and *β-actin* were used as loading controls.
